# Antitumor Effect of Anti‐c‐Myc Aptamer‐Based PROTAC for Degradation of the c‐Myc Protein

**DOI:** 10.1002/advs.202309639

**Published:** 2024-04-29

**Authors:** Yuchun Wang, Gang Yang, Xinyu Zhang, Ruoling Bai, Deyu Yuan, Denghui Gao, Qianyu He, Yi Yuan, Xinghe Zhang, Junchuang Kou, Lihua Zheng, Yanxin Huang, Zhuo Tang, Yongli Bao, Xu Song, Yongyun Zhao

**Affiliations:** ^1^ National Engineering Laboratory for Druggable Gene and Protein Screening College of Life Science Northeast Normal University Changchun 130024 P. R. China; ^2^ College of Life Science Sichuan University Chengdu Sichuan 610064 P. R. China; ^3^ Natural Products Research Centre Chengdu Institution of Biology Chinese Academy of Science Chengdu 610041 P. R. China; ^4^ lncTAC Bio. Chengdu Sichuan 610200 P. R. China

**Keywords:** aptamer‐based PROTAC, c‐Myc aptamer, c‐Myc degradation, microwell‐SELEX, undruggable

## Abstract

Targeting “undruggable” targets with intrinsically disordered structures is of great significance for the treatment of disease. The transcription factor c‐Myc controls global gene expression and is an attractive therapeutic target for multiple types of cancers. However, due to the lack of defined ligand binding pockets, targeted c‐Myc have thus far been unsuccessful. Herein, to address the dilemma of lacking ligands, an efficient and high throughput aptamer screening strategy is established, named polystyrene microwell plate‐based systematic evolution of ligands by exponential enrichment (microwell‐SELEX), and identify the specific aptamer (MA9C1) against c‐Myc. The multifunctional aptamer‐based Proteolysis Targeting Chimeras (PROTAC) for proteolysis of the c‐Myc (ProMyc) is developed using the aptamer MA9C1 as the ligand. ProMyc not only significantly degrades c‐Myc by the ubiquitin‐proteasome system, but also reduces the Max protein, synergistically inhibiting c‐Myc transcriptional activity. Combination of the artificial cyclization and anti‐PD‐L1 aptamer (PA1)‐based delivery system, circular PA1‐ProMyc chimeras achieve tumor regression in the xenograft tumor model, laying a solid foundation for the development of efficacious c‐Myc degrader for the clinic. Therefore, this aptamer‐based degrader provides an invaluable potential degrader in drug discovery and anti‐tumor therapy, offering a promising degrader to overcome the challenge of targeting intractable targets.

## Graphical abstract

The drugs against “Undruggable” targets remain an enormous challenge in drug discovery. Aptamers are used to address the ligand deficiency and isolate the anti‐c‐Myc aptamer. The aptamer‐based PROTAC that efficiently degrades c‐Myc (ProMyc) is developed. Based on the aptamer (PA1) delivery system, circular PA1‐ProMyc displays powerful antitumor activity in the tumor model, offering a potential degrader for intractable targets.

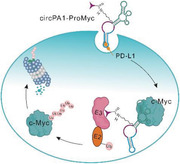

## 1 Introduction

The c‐Myc protein, an oncogenic transcription factor, is considered to be an effective target for cancer therapy as it is associated with up to 70% of human cancers and plays critical roles in tumorigenesis and therapeutic resistance.^[^ ^1^ ^]^ Nevertheless, there are several conceptual and practical difficulties in targeting the c‐Myc protein which is regarded as the traditionally “undruggable” target, including an intrinsically disordered structure, lacking a defined pocket for small‐molecule binding, localization in the nucleus and potential “on‐target” toxicity to normal tissues.^[^ ^2^ ^]^ Among them, the intractable targets function through protein‐protein interaction (PPI) interfaces and have no definite 3D structure in a free state, which is a big challenge in screening their small molecule binders.^[^ ^2a^ ^]^ Moreover, targeting the nuclear c‐Myc with specific antibodies is very difficult. To date, a dominant‐negative mutant Myc‐inhibiting peptide (OmoMYC) that binds directly to c‐Myc and abrogates c‐Myc function was conducted in a phase I clinical trial.^[^ ^3^ ^]^ Currently, attempts to target c‐Myc directly have focused on the c‐Myc /Max heterodimerization domain. Several small‐molecule inhibitors (e.g., EN4, IIA6B17, MYCi975) disrupt c‐Myc /Max heterodimer.^[^ ^4^ ^]^ In addition to direct inhibition, there are indirectly regulating the upstream and downstream pathways of c‐Myc, such as targeting Max monomers and homodimers (KI‐MS2‐008, NSC13728),^[^ ^5^ ^]^ c‐Myc stability.^[^ ^6^ ^]^ Additionally, unlike RAS and p53, the abnormality of c‐Myc is overexpression in tumors and downregulation of c‐Myc mRNA by ASO or siRNA to directly inhibit c‐Myc expression is another strategy.^[^ ^7^ ^]^ Nevertheless, despite so many strategies that have been developed, the challenge is the current absence of a clinically viable c‐Myc inhibitor.^[^ ^1^, ^6^ ^]^ Nevertheless, screening the anti‐c‐Myc ligands and development of drugs against c‐Myc remains an enormous challenge in drug discovery and anti‐tumor therapy.

PROTAC technology which is superior to conventional small molecule inhibitors has significantly advanced during the last decade. PROTAC consists of a small molecule of the protein of interest (POI) ligand, a linker, and an E3 ligase ligand, which recruits that E3 ligase to the POI to induce the ubiquitination, leading to its degradation via the proteasome pathway.^[^ ^8^ ^]^ PROTACs trigger the destruction of POIs in an event‐driven manner, providing a new promising modality to inhibit previously undruggable targets.^[^ ^9^ ^]^ However, small‐molecule PROTAC approaches are difficult to target undruggable proteins without efficient small‐molecule binders. Recently, oligonucleotide‐based PROTAC which use oligonucleotide motifs to substitute small molecules as POI ligands have developed, such as RNA‐PROTACs,^[^ ^10^ ^]^ O'PROTAC,^[^ ^11^ ^]^ TF‐PROTAC,^[^ ^12^ ^]^ TRAFTAC,^[^ ^13^ ^]^ OligoTRAFTACs.^[^ ^14^ ^]^ Those PROTACs that target the degradation of transcription factors (TFs) take advantage of the intrinsic DNA binding ability of TFs and use double‐stranded DNA as the POI ligand. Nevertheless, their binding affinity to the target may not be sufficient.^[^ ^15^ ^]^

Aptamers, also known as chemical antibodies, can bind to a wide range of targets, including proteins, small metal ions, viruses, bacteria, cells, and so on. They can even distinguish closely related molecules such as conformational isomers, or amino acid mutations.^[^ ^16^ ^]^ Compared with small molecule ligands which require specific binding pockets on the target, the aptamers basically have no special requirement for their target. Moreover, compared with antibodies, it is small in size to target a variety of protein formats. Therefore, aptamers which are used as the chemical biology tool show great advantage in targeting undruggable proteins to address the dilemma of lacking ligands or the nuclear localization. Recently, some studies have exploited aptamers as ligands in aptamer‐based PROTAC.^[^ ^17^ ^]^ Among them, TNA (threose nucleic acid) aptamers targeting the c‐Myc/Max dimer are conjugated to E‐box (double‐stranded DNA), and the conjugates act as a ligand in PROTAC (TNA‐E box‐pomalidomide, TEP) to degrade c‐Myc/Max dimer.^[^ ^18^ ^]^ However, single TEP is ineffective in tumor suppression in mice.

Traditional aptamer selection generally requires the protein with a tag, and involves issues with success rate and nonspecific binding.^[^ ^19^ ^]^ Here, inspired by the enzyme‐linked immunosorbent assay (ELISA), we exhibit a novel polystyrene microwell‐based selection platform (microwell‐SELEX) which was suitable for high‐throughput aptamer selection with high selectivity, especially for untagged protein. Using this technology, the aptamer MA9C1 specifically binds to c‐Myc was identified. We construct the multifunctional aptamer‐based PROTAC for proteolysis of the c‐Myc (ProMyc) and demonstrate that ProMyc is also a bridged PROTAC that also significantly reduces the Max protein, remarkably inhibiting c‐Myc transcriptional activity. Furthermore, the artificial cyclization and anti‐PD‐L1 aptamer (PA1) with the delivery function that we have reported were introduced to increase stability and efficient delivery.^[^ ^20^ ^]^ The circular PA1‐ProMyc (circPA1‐ProMyc) potently suppresses tumorigenesis in xenografted tumor mice, providing a potential degrader for drug discovery targeting c‐Myc.

## 2 Results and Discussion

### 2.1 Identification and Performance of Aptamer Against c‐Myc Screened by Microwell‐SELEX

**Figure 1:**
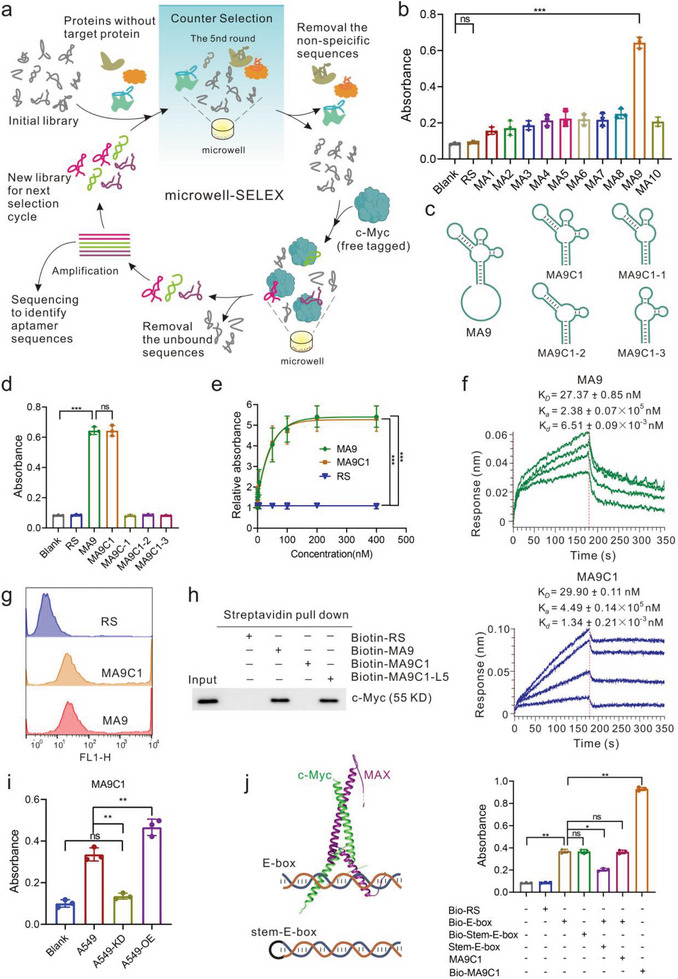
Identification and Performance of aptamers screened by microwell‐SELEX. a) The scheme of microwell‐SELEX that coated proteins on plates for the discovery of aptamers against the untagged c‐Myc protein. b) Identification of candidate sequences of biotin‐labeled aptamer (200 nM) with a high abundance in enriched pools to bind to c‐Myc (100 ng/well) pre‐coated on microplates. c) Structure of the truncated aptamers according to the MA9. d) ELISA analysis of the binding performance of the truncated aptamers. e) ELISA analysis of the dose‐dependent binding of the aptamers MA9 and MA9C1 to c‐Myc protein. f) Bio‐Layer Interferometry assay for the kinetic binding parameters of aptamer binding to immobilized c‐Myc. g) Flow cytometry analysis of the binding performance of FAM‐labeled aptamer (MA9, MA9C1) (200 nM) to His‐tagged c‐Myc (His‐c‐Myc) that purified from HEK‐293T cells with overexpressing His‐c‐Myc. Random sequences were used as baseline controls. h) Streptavidin pull‐down assay analysis of the aptamer binding with c‐Myc protein in cell lysates overexpressing c‐Myc. i) The selectivity of aptamer binding to A549 cell lysates with different treatments. OE: overexpressing c‐Myc with the transfection of His‐c‐Myc plasmid; KD: cells transfected with c‐Myc ASO for 48 h to silence c‐Myc. j) Competitive ELISA analysis of the effect of aptamer MA9C1 on the binding of c‐Myc/Max dimer to E‐box. All the error bars indicate standard deviations (*n* = 3). All the *p* values were determined. ^*^ *p < *0.05, ^**^ *p < *0.01, ^***^ *p < *0.001.

At present, the traditional method of screening aptamer for protein is usually magnetic beads‐SELEX, which is relatively simple but requires the tagged protein that is easy to cause non‐specific binding. To eliminate the possible interference of tags, it is preferable to screen with untagged protein. Inspired by the ELISA, here, a novel selection platform, microwell‐SELEX, for untagged protein was established (**Figure** **1**a). Briefly, the untagged c‐Myc protein produced by the mammalian expression system was used as the target. The synthetic ssDNA library was incubated with c‐Myc pre‐coated on a microwell plate for SELEX. To further reduce nonspecific bounding DNA, after 6 rounds of selection, total protein in HCT116 cells with the c‐Myc knockdown was used as the negative targets to conduct counter‐selection. After 12 rounds of selection, the final aptamer pool products were assessed by next‐generation sequencing and the 10 most abundant sequences were selected as candidate sequences (Figure S1a,b, Supporting Information).

Next, the binding performance of those candidate sequences was evaluated as we reported previously.^[^ ^21^ ^]^ MA9 exhibited the highest affinity (Figure 1b). Subsequently, we predicted the structure of the aptamer by Nupack software and removed the free‐end sequences, resulting in a cloverleaf‐structured aptamer MA9C1 (59 nt). For further truncation, a series of two‐leaf structures were obtained (Figure 1c). Among them, MA9C1 exhibits an excellent binding capacity comparable to full‐length aptamer MA9 (Figure 1d,e). And the binding kinetics were measured by biolayer interferometry assay (BLI). The dissociation constants (Kd) for aptamers binding to c‐Myc are 27.37 nM and 29.90 nM, respectively (Figure 1f). Together, those aptamers against c‐Myc exhibit high binding affinity, further indicating that a screening method for aptamers against unlabeled proteins has been established and microwell‐SELEX is a high‐throughput screening platform.

Due to the differences between in vitro and intracellular environments, we evaluated the ability of aptamers to bind to intracellular c‐Myc. We overexpressed and purified His‐tagged c‐Myc protein (Figure S1c, Supporting Information). The flow cytometry analysis and fluorescent images showed that those aptamers have a strong binding capacity(Figures 1g and S1d, Supporting Information). To further confirm this result, we performed the streptavidin pull‐down experiment to determine whether aptamers are capable of binding to intracellular c‐Myc (Figure 1h). Unexpectedly, aptamer MA9C1 is unable to bind to c‐Myc. We suspected that it is the modification of biotin at the 5‐terminal of MA9C1 that decreases the binding efficiency, and we added the extra five bases between biotin and the MA9C1 (MA9C1‐L5) to prove this hypothesis. Notably, fluorescence images and the 3D projections of Z‐stack image imaging of the localization of cy3‐labeled MA9C1 verified that the red signals embedded in the Hoechst‐stained nuclei, demonstrating that the MA9C1 could be localized in the nucleus which is consistent with the nuclear localization of c‐Myc (Figure S1e–g, Supporting Information). The random sequence (RS) was mainly located in the cytoplasm, indicating that the MA9C1 may follow the c‐Myc protein into the nucleus.

Additionally, selectivity is another very important performance. A549 cells with c‐Myc knockdown (A549‐KD) or overexpression (A549‐OE) were taken as the negative and positive control, respectively. The ELISA results show that the A549‐OE displays the highest fluorescence, while the value of A549‐KD was significantly decreased (Figure 1i; Figure S2a, Supporting Information). Furthermore, the total proteins of HCT116 cells were incubated with biotin‐labeled MA9. The binding complex was then separated using streptavidin‐coated magnetic beads and analyzed through SDS‐PAGE gel. Compared with the random sequence, one distinguishing band with a relative molecular mass of ≈60 kDa was present (Figure S2b, Supporting Information). This characteristic band was then submitted to LC‐MS analysis and produced a list of protein hits (Table S1, Supporting Information). As expected, among these candidates, c‐Myc ranked first with the maximum content. Those results indicated that aptamer has ideal selectivity, providing the potential ligand against c‐Myc.

c‐Myc is a disordered protein and directly disrupts c‐Myc/Max dimer or inhibits DNA binding are the widely used strategies.^[^ ^6^ ^]^ Molecular docking simulations provide that four nucleobases are involved in the formation of a hydrogen‐bonding network with c‐Myc, and that the aptamer MA9C1 binds to an intrinsically disordered region of c‐Myc, distinct from the epitope bound to Max (Figure S2c, Supporting Information). Sequences with mutations in the predicted binding sites (MA9C1‐mut) fail to bind c‐Myc (Figure S2d, Supporting Information) to confirm the molecular docking model. And competitive ELISA results showed that aptamer MA9C1 did not block c‐Myc/Max interaction (Figure S2e, Supporting Information). Additionally, we also assessed whether MA9C1 affects the c‐Myc/Max dimer to bind to DNA. The E‐box sequence (E‐Box, 5′‐CACGTG‐3′ ) which was a well‐known binding motif^[^ ^1b^ ^]^ for c‐Myc/Max was used. Meanwhile, to reduce the interference of double‐stranded DNA annealing, we also designed a single‐stranded DNA (stem‐E box) that can fold itself into a stem‐loop structure containing the E‐Box. The results show that aptamer MA9C1 does not interfere with c‐Myc/Max dimer binding to E‐box. Notably, aptamer MA9C1 shows stronger affinity than E‐box (Figure 1j). Those demonstrate that MA9C1 is biologically inert and binds to the intrinsically disordered region.

### 2.2 Design and Characteristics of the Degrader for c‐Myc Degradation

**Figure 2:**
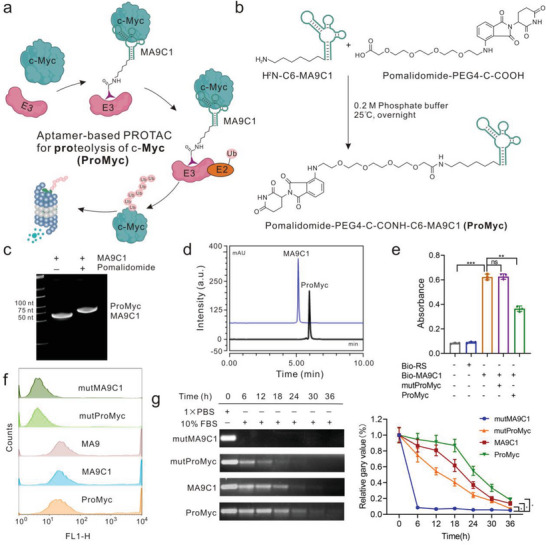
Design and characteristics of the aptamer‐based PROTAC for c‐Myc. a) The schematic diagram depicts the strategy of aptamer‐based PROTAC for c‐Myc (ProMyc) to mediate proteasomal degradation of the “undruggable” transcription factors. b) Pomalidomide‐PEG4‐C‐COOH is linked to the C6‐aptamer MA9C1 (NH2‐C6‐MA9C1) via an amide bond to form ProMyc. c) 20% native PAGE analysis of the incorporation of Pomalidomide onto the aptamer MA9C1. d) HPLC identifies the ProMyc purification. e and f) Competition ELISA assay e) and flow cytometry f) analysis of the binding performance of ProMyc (200 nM) to c‐Myc protein (50 ng). g) Stability and the quantitative analysis of ProMyc (500 ng) incubated in 10% FBS at 37 °C at the indicated time. All the error bars indicate standard deviations (*n* = 3). All the *p* values were determined. ^*^ *p < *0.05, **p < 0.01, ^***^ *p < *0.001.

The schematic diagram of the anti‐c‐Myc aptamer‐based PROTAC is illustrated in **Figure** **2**a. The aptamer MA9C1 with NH_2_‐C6 modification at the 5′ end was conjugated with the molecule ligand (pomalidomide‐PEG4‐COOH) of E3 ubiquitin ligase cereblon (CRBN), to generate the c‐Myc degrader (ProMyc) (Figure 2b). This dehydration condensation reaction was analyzed by 20% native PAGE which exhibited that the molecular weight will increase when pomalidomide‐PEG4‐COOH is incorporated into NH2‐C6‐MA9C1(Figure 2c) and the purified products were also identified by HPLC and mass spectrometry(Figure 2d; Figure S3a,b, Supporting Information). The mutants of aptamer, which has no affinity for c‐Myc, were also linked to pomalidomide as a negative control (mutProMyc).

Subsequently, the binding performance of ProMyc was evaluated and the results indicated that the pomalidomide in ProMyc did not affect the binding capacity (Figure 2e). Likewise, the flow cytometry analysis and fluorescent images further confirmed the performance of ProMyc binding to intracellular c‐Myc (Figure 2f; Figure S3c, Supporting Information). The fluorescence microscopy imaging also verified that ProMyc was partially localized in the nucleus which was highly consistent with that of the individual aptamer MA9C1(Figure S3d, Supporting Information). Additionally, one of the difficulties associated with oligonucleotides is their rapid degradation. We evaluated their stability and found that the cloverleaf‐structured aptamer MA9C1 has certain stability. And fortunately, compared with the aptamer MA9C1, the stability of ProMyc is significantly improved (Figure 2g). We suspected this was due to the small molecule preventing the 5′ exonuclease from degrading the aptamer MA9C1. Together, the connection of aptamer with small molecule ligand does not affect the binding capacity and contributes the stability.

### 2.3 ProMyc Induces the Degradation of c‐Myc in Cells

**Figure 3:**
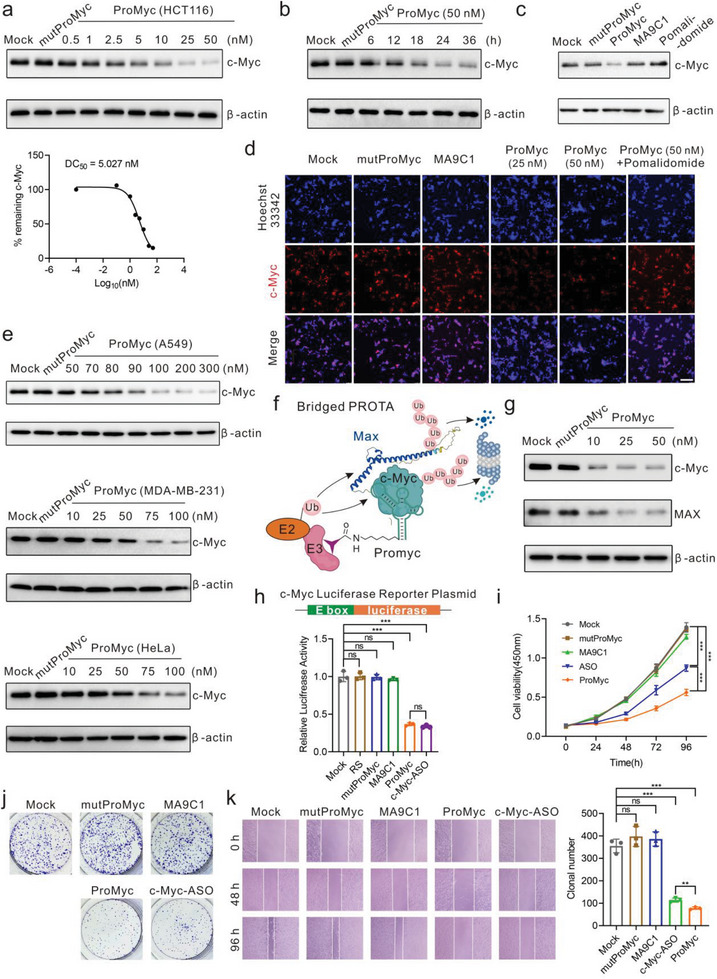
ProMyc effectively degrades the endogenous c‐Myc and inhibits cell proliferation and tumorigenesis. a) Representative western blot analysis of the c‐Myc protein in HCT116 cells transfected with different doses of ProMyc for 24 h. The concentration of mutProMyc is 50 nM in HCT116 cells. The remaining c‐Myc (%) was calculated by normalizing the value in each group to that in the Mock and the DC_50_ was determined. b) HCT116 cells were transfected with ProMyc (50 nM) for the indicated time points, followed by western blot analysis of the c‐Myc protein. c) Western blot analysis of the c‐Myc protein in HCT116 cells treated with ProMyc (50 nM), MA9C1 (50 nM), pomalidomide (10 µM)), mutProMyc (50 nM), respectively. d) Immunofluorescence images of c‐Myc in HCT116 cells after 24 h treatment of ProMyc. Red signals indicate c‐Myc. Scale bar, 50 µm. e) Representative western blot analysis of the c‐Myc protein in various cells (A549, MDA‐MB‐231, HeLa) transfected with different concentrations of ProMyc for 24 h. The concentration of mutProMyc is the maximum concentration of ProMyc. f) The schematic diagram depicts the strategy of bridged PROTAC for c‐Myc and Max to mediate proteasomal degradation. g) Representative western blot analysis of the c‐Myc and Max protein in HCT116 cells treated with the indicated concentration of ProMyc for 24 h. h) c‐Myc luciferase reporter activity in HCT116 cells cotransfected MYC luciferase reporter plasmid and ProMyc (50 nM) for 48 h. c‐Myc ASO: the positive control. i) Identify the cell proliferation inhibition in HCT116 cells with the ProMyc (50 nM) treatment at the indicated times by CCK8 assay. c‐Myc ASO: the positive control. j) Representative image analysis of the inhibition of ProMyc on HCT116 cell migration and tumorigenicity in the colony formation assay. k) The inhibition of ProMyc on HCT116 cell migration and tumorigenicity in wound‐healing assay. Left: representative images. Right: the quantification results. All the oligonucleotide concentrations were 50 nM. All the error bars indicate standard deviations (*n* = 3). All the *p* values were determined. n.s., not significant; ^***^ *p* < 0.001.

ProMyc exhibits excellent degradation of c‐Myc protein at 50 nM with a maximum degradation (D_max_) of 95% and the DC_50_ (50% degradation) of 5.02 nM in HCT116 cells transfected with ProMyc for 24 h (**Figure** **3**a). Furthermore, ProMyc degrades c‐Myc protein in a time‐dependent manner (Figure 3b). In contrast, the mutProMyc is also ineffective in c‐Myc degradation. Likewise, neither MA9C1 nor pomalidomide had any effect on c‐Myc (Figure 3c). To further confirm this result, we also used immunofluorescent staining to identify the c‐Myc protein in situ. HCT116 cells transfected with ProMyc showed a significant decrease in fluorescence and the fluorescence intensity was negatively correlated with the dose of ProMyc (Figure 3d). Moreover, ProMyc‐induced c‐Myc degradation in other relevant tumor cells (A549, HeLa, and ME‐MB‐231) in a dose‐dependent and time‐dependent manner (Figure 3e; Figure S4a,b, Supporting Information) and the DC_50_ was 98.52 nM in A549 cells (Figure S4c, Supporting Information). ProMyc had a weak effect on the levels of c‐Myc homologous proteins (Figure S4d, Supporting Information). This may be due to the fact that aptamer binds to amino acid segments, which are distinct in those proteins (Figure S4e, Supporting Information). Additionally, we suspected that c‐Myc acts as a bridge PROTAC^[^ ^22^ ^]^ to promote the degradation of Max (the half‐life ≈24 h) (Figure 3f). As expected, the Max associated with tumorigenesis is significantly reduced (Figure 3g).

The length of the linker between the POI ligand and E3 ligand is an important factor for the effective destruction of the target protein. Herein, we wondered whether the linker of ProMyc could also influence the degradation efficiency. The aptamer MA9C1 with variants linker (0A, 5A, 10A, 15A) was conjugated with pomalidomide, named ProMyc‐LX, respectively (FigureS5a,b, Supporting Information). There was no significant difference in the affinity of ProMyc‐LX with different linkers (Figure S5c, Supporting Information). Among them, ProMyc without A afforded relatively better results to downregulate the c‐Myc (Figure S5d, Supporting Information), suggesting that it is necessary to optimize the length of the linker between the aptamer and E3 ligand. Otherwise, OligoTRAFTACs demonstrated that VHL ligand and c‐Myc‐binding oligonucleotide could degrade c‐Myc.^[^ ^14^ ^]^ The aptamer MA9C1 was conjugated with VHL ligand (AHPC) to construct ProMyc‐VHL (Figure S6a, Supporting Information). Likewise, PAGE, HPLC, and mass spectrometry were used to identify the ligation product (Figure S6b,c, Supporting Information). The affinity of ProMyc‐VHL is highly consistent with ProMyc (Figure S6d, Supporting Information). Compared to the degradation efficiency of different E3 ligands, CRBN was superior to VHL (Figure S6e, Supporting Information).

Next, we ask whether the reduction of c‐Myc would affect c‐Myc‐regulated transcriptional targets. The c‐Myc luciferase reporter assay was performed using a c‐Myc‐dependent E‐box luciferase reporter. The cells co‐transfected with the c‐Myc luciferase reporter and ProMyc exhibit quite lower luciferase activity, indicating that ProMyc remarkably inhibits the c‐Myc transcriptional activity (Figure 3h; Figure S7a, Supporting Information). And the ability of ProMyc to reduce luciferase activity was comparable to that of the c‐Myc inhibitor (MYCi361) (Figure S7b, Supporting Information). Previously reported that the cell cycle is strongly related to c‐Myc.^[^ ^23^ ^]^ Hence, FACS analysis of the cell cycle showed that the number of cells dramatically decreased in the S‐phase, accompanied by an increase in the G_1_ and G_2_/M, indicating that c‐Myc downregulation impairs cell cycle progression (Figure S7c, Supporting Information). To further confirm this conclusion, qPCR analysis shows that the RNA associated with cell cycle regulation (CDK4, CDC25, CDK2) is reduced (Figure S7d, Supporting Information) which is consistent with the previous reports.^[^ ^24^ ^]^ Collectively, ProMyc could significantly decrease c‐Myc protein and effectively inhibit its transcriptional activity.

We further explored the effects of ProMyc on cell proliferation and migration. Compared with the knockdown c‐Myc, the cells transfected with ProMyc dramatically inhibited the proliferation (Figure 3i). Consistently, ProMyc notably inhibited migration (Figure 3j; Figure S7e, Supporting Information) and tumorigenesis (Figure 3k), while the individual aptamer MA9C1 has no anti‐tumor activity. Additionally, transfection with a high concentration of aptamer MA9C1 does not affect the cell viability to eliminate the cytotoxicity (Figure S7f, Supporting Information). Together, those demonstrated that ProMyc is capable of degrading c‐Myc and affects the downstream targets, ultimately impairing tumorigenesis.

### 2.4 ProMyc Recruits E3 Ubiquitin Ligase to Degrade c‐Myc via the Proteasomal Pathway

**Figure 4:**
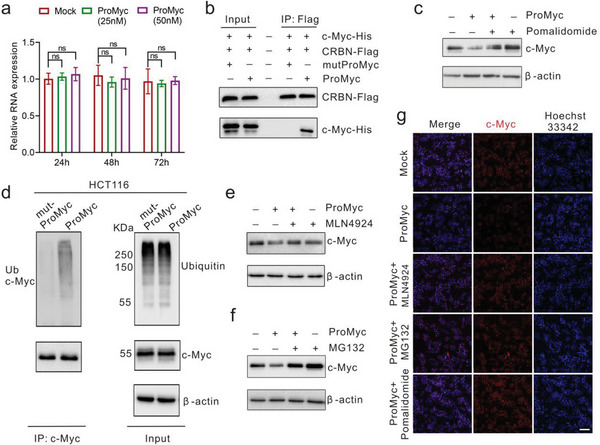
ProMyc redirects E3 ubiquitin ligase to degrade c‐Myc via the proteasomal pathway. a) qRT‐PCR analyses of *c‐Myc* mRNA in HCT116 cells with ProMyc treatment at the indicated time. b) The immunoprecipitation with anti‐Flag or anti‐His antibodies to analyze the interaction of c‐Myc and CRBN in the lysates of HEK‐293T cells co‐transfected with plasmids expressing Flag‐CRBN and His‐c‐Myc. The cells were incubated with MG132 (20 µM) for 1 h, followed by treatment with ProMyc (50 nM) for 12 h. c) c‐Myc protein levels in HCT116 cells treated with ProMyc in the presence of Pomalidomide. The cells were incubated with Pomalidomide (10 µM) for 1 h, followed by treatment with ProMyc (50 nM) for 24 h. d) Western blotting for the ubiquitination of c‐Myc in HEK‐293T cells with the ProMyc treatment. Cells transfected with plasmids expressing HA‐Ub were incubated with MG132 (20 µM) for 1 h, followed by treatment with ProMyc (50 nM) for 12 h. c‐Myc was immunoprecipitated with anti‐Myc antibody, and the ubiquitination was detected with the anti‐Ub antibody. e and f) c‐Myc protein levels in HCT116 cells treated with ProMyc in the presence of proteasome inhibitor MLN4924 e) or proteasome inhibitor MG132 f). The cells were incubated with MLN4924 (20 µM) or MG132(20 µM) for 1 h, followed by treatment with ProMyc (50 nM) for 24 h. g) Immunofluorescence images of c‐Myc protein in HCT116 cells. The cells were treated with ProMyc (50 nM) in the presence of Pomalidomide (10 µM). The cells were incubated with MLN4924 (20 µM), MG132 (20 µM) and Pomalidomide (10 µM) for 1 h, respectively, and then treated with ProMyc (50 nM) for 24 h. Scale bar, 50 µm.

Having identified ProMyc as a potent c‐Myc degrader, we wondered whether the reduction of c‐Myc was through the expected degradation mechanism of the ubiquitin‐proteasome system (UPS). We found that ProMyc had no impact on the levels of *c‐Myc* mRNA, suggesting the effect of ProMyc on c‐Myc protein reduction at the post‐translational level (**Figure** **4**a). Immunofluorescence results showed that CRBN was distributed in both the nucleus and cytoplasm and mainly located in the nucleus, indicating that CRBN has the advantage of geographical proximity to the c‐Myc protein (Figure S8a, Supporting Information). We determined whether ProMyc could promote the interaction between c‐Myc and CRBN. The lysates of cells transfected with ProMyc were prepared to conduct co‐immunoprecipitation (Co‐IP) using an anti‐c‐Myc antibody. As expected, CRBN was present in c‐Myc immunoprecipitants only in the presence of ProMyc (Figure 4b), indicating that ProMyc recruits CRBN E3 ubiquitin ligase to c‐Myc to mediate their ternary complex formation. Moreover, the ProMyc‐induced degradation of the c‐Myc was effectively blocked by pomalidomide (Figure 4c), implying that pomalidomide disrupted their ternary complex formation.

Next, when the cells were co‐transfected with the plasmid expressing an HA‐tagged ubiquitin (HA‐Ub) and His‐tagged c‐Myc (His‐c‐Myc) plasmid, ProMyc significantly increased the c‐Myc ubiquitination (Figure 4d). Furthermore, the ubiquitination of c‐Myc protein could be suppressed after cells were treated with MLN4924, the cullin neddylation inhibitor to inhibit the ubiquitination (Figure 4e). Consistently, the ProMyc‐induced decrease in c‐Myc protein was also abolished by a proteasome inhibitor, MG132 (Figure 4f). Moreover, overexpression of CRBN aggravated the c‐Myc degradation, while knockdown of the CRBN prevented the degradation to further confirm the dependence of c‐Myc degradation on CRBN and ubiquitin modification (Figure S8b–d, Supporting Information). The immunofluorescence images further confirm this result that it is the proteasome degradation pathway to degrade c‐Myc (Figure 4g). Overall, those results indicate that the reduction of c‐Myc was through the expected degradation mechanism of the ubiquitin‐proteasome system (UPS).

### 2.5 The Antitumor Potential of ProMyc in Mouse Xenograft Tumors

**Figure 5:**
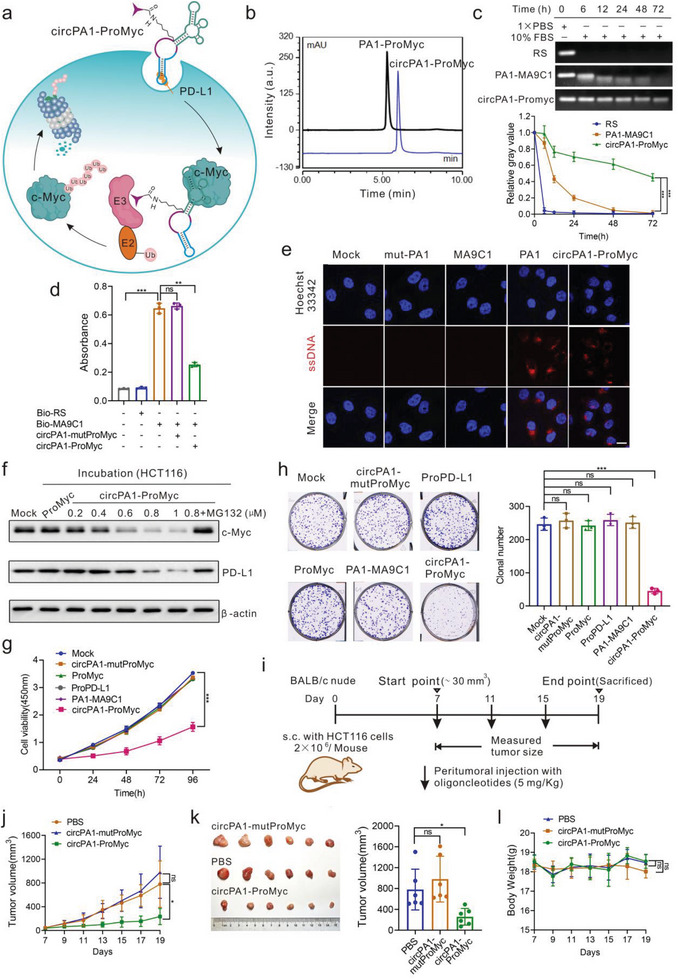
The antitumor potential of ProMyc in vitro and xenograft tumors. a) The principle of circPA1‐ProMyc delivery system to targeted degradation of the c‐Myc protein in cells. b) HPLC identifies the purification of the incorporation of Pomalidomide onto the circPA1‐MA9C1. c) The stability of circPA1‐ProMyc incubated in the DMEM medium containing 10% FBS at 37 °C. d) Competitive ELISA analysis of the performance of circPA1‐ProMyc binding to c‐Myc protein. The concentration of indicated oligonucleotide is 100 nM. e) Confocal microscopy imaging of HCT116 cells to examine the internalization of circPA1‐ProMyc. The cells were incubated with circPA1‐ProMyc (500 nM) for 1 h at 37 °C. Scale bar, 10 µm. f) Representative western blot analysis of the c‐Myc and PD‐L1 in HCT116 cells incubated with different concentrations of circPA1‐ProMyc for 24 h. The concentration of ProMyc used to incubate HCT116 cells is 800 nM. g) Anti‐proliferative effects of circPA1‐MA9C1 on HCT116 cells following 4 days of treatment. The cells were incubated with different oligonucleotides (500 nM) and cell survival was determined with a CCK‐8 kit. h) Colony formation assay analysis of the anti‐tumorigenesis activity of circPA1‐MA9C1 on HCT116 cells. The cells were incubated with different oligonucleotides (500 nM) for 8 days. i) HCT116 tumor‐bearing mice (*n* = 6 mice /group) were peritumorally administered circPA1‐MA9C1 (5 mg k^−1^g) or PBS every 4 days on the 7th day after tumor inoculation. j) Tumor volumes of HCT116 xenograft tumors from the indicated groups of mice after treatment with circPA1‐ProMyc or PBS were recorded on the indicated days (*n* = 6 mice /group). k) Photos and the tumor volumes of HCT116 xenograft tumors at day 19 after the indicated treatment. l) The body weight of mice was measured at the indicated time points after the treatments in xenograft (*n* = 6 mice /group). *p* values were determined. n.s., not significant; ^*^ *p < *0.05, ^**^ *p < *0.01, ^***^ *p < *0.001.

To improve the pharmaceutical potential of ProMyc, the artificial cyclization and anti‐PD‐L1 aptamer (PA1) with the delivery function that we have reported were introduced to increase stability and targeted delivery.^[^ ^20^ ^]^ We employed the anti‐PD‐L1 aptamer PA1 as the delivery tool and constructed the circular PA1‐ProMyc (circPA1‐ProMyc) chimera which can internalize into the PD‐L1 positive cells. The expected schematic diagram of circPA1‐ProMyc is illustrated in **Figure** **5**a. ProMyc could be effectively delivered into cells to degrade c‐Myc protein. The circPA1‐ProMyc was identified by HPLC and MS (Figure 5b; Figure S9a, Supporting Information). The stability of the circPA1‐ProMyc is superior to that of the linear chimera in serum (Figure 5c). Moreover, the performance of circPA1‐ProMyc to bind to c‐Myc was highly consistent with that of the individual aptamer (Figure 5d). Meanwhile, cy3‐labeled circPA1‐ProMyc was incubated with HCT116 cells and the chimera was successfully delivered into cells (Figure 5e). As expected, the c‐Myc was significantly decreased in the cells incubated with circPA1‐ProMyc (Figure 5f) and the same result was also found in cells transfected with circPA1‐ProMyc (Figure S9b, Supporting Information). Interestingly, the PD‐L1 was also significantly decreased. We speculated that this degradation is through a dual mechanism. On the one hand, PD‐L1 is the downstream regulatory target of c‐Myc^[^ ^25^ ^]^ and qRT‐PCR analysis also verified that the c‐Myc degradation was beneficial to the down‐regulation of PD‐L1 (Figure S9c, Supporting Information). On the other hand, circPA1‐ProMyc may be a dual aptamer‐based PROTAC for the simultaneous degradation of c‐Myc and PD‐L1. Hence, to test this, we synthesized the aptamer PA1 conjugated with pomalidomide (ProPD‐L1) and found that ProPD‐L1 could effectively decrease PD‐L1(Figure S9d, Supporting Information). Furthermore, circPA1‐mutProMyc only decreases PD‐L1 but not c‐Myc, whereas circPA1‐ProMyc can reduce both proteins (Figure S9e, Supporting Information). Flow cytometry analysis further confirms that circPA1‐ProMyc is superior to degrade PD‐L1(Figure S9f, Supporting Information). Those demonstrate that circPA1‐ProMyc is a dual aptamer‐based PROTAC.

Furthermore, the proliferation of HCT116 cells incubated with circPA1‐ProMyc was significantly inhibited, which was highly consistent with that of transfection (Figure 5g; Figure S10a, Supporting Information). Moreover, inhibition of proliferation potential was dose‐dependent in the concentration range from 0.4 µM to 1 µM (Figure S10b, Supporting Information). circPA1‐ProMyc also efficiently reduced the migration and tumorigenesis of HCT116 cells (Figure 5h; Figure S10c, Supporting Information). To answer whether this antitumor activity is related to the PD‐L1 degradation, ProPD‐L1 was used as the control. We found that the degradation of PD‐L1 alone did not affect cell proliferation and tumorigenesis, confirming that the antitumor activity of circPA1‐ProMyc is due to the c‐Myc degradation.

After establishing the efficacy of circPA1‐ProMyc in cells, we wondered whether circPA1‐ProMyc would exhibit the antitumor potential in *vivo*. HCT116 colorectal tumor xenograft in immune‐deficient mice (BALB/c Nude mice) was established. Those mice were treated with circPA1‐ProMyc at 5 mg k^−1^g by peritumoral injection, every 4 days from day 8 to day 20 day (Figure 5i). On day 19, all the animals were sacrificed and the tumors were photographed and dissected. As expected, circPA1‐ProMyc remarkably attenuated tumor growth compared to the control PBS‐treated mice (Figure 5j,k). Those mice showed no significant weight loss (Figure 5l). Moreover, consistent with the effect of circPA1‐ProMyc in cells, circPA1‐ProMyc treatment significantly decreased c‐Myc protein in tumors (Figure S10d, Supporting Information). Overall, circPA1‐ProMyc degradats the c‐Myc and exhibits potent efficacy in antitumor therapy.

Additionally, we also evaluated the cytotoxicity of this circPA1‐ProMyc, the CCK‐8 assay was performed and indicated no significant effects on the viability of HEK‐293T cells (Figure S10e, Supporting Information). Furthermore, this circPA1‐ProMyc was dissolved in the physiological saline solution and administered intraperitoneally to C57BL/6 mice to evaluate its hepatotoxicity and immunogenicity. The levels of inflammatory cytokines (TNF‐a and IL‐6) display that circPA1‐ProMyc has low immunogenicity (Figure S10f, Supporting Information). Together, those demonstrate the effectiveness and safety of circPA1‐ProMyc in therapy.

## 3 Conclusion

Target occupancy is not sufficient to elicit biological activity, particularly for the difficult‐to‐drug targets. Small‐molecule‐based PROTACs have shown great promise in the clinic. Nevertheless, there are longstanding challenges surrounding the ligand insufficient for “undruggable” protein targets. Here, we advocate that aptamer can be the source of ligands. The novel microwell‐SELEX used for aptamer selection not only can be used for the untagged protein with high selectivity but also provides a high‐throughput technique. Compared with the natural ligand E‐box, the aptamer MA9C1 against c‐Myc is superior in affinity and stability. The aptamer also overcomes the difficulty of targeting c‐Myc due to its nuclear localization. We put forward that aptamers are powerful tools to solve ligand deficiency.

No attempt should be left aside to overcome the challenge of degradation of the undruggable c‐Myc. The ProMyc selectively targeting the c‐Myc for proteasome‐mediated degradation is developed. In terms of oligonucleotide‐based PROTACs, compared with the double‐stranded DNA which bears a well‐defined duplex structure,^[^ ^11^ ^]^ the aptamer is desirable for drug development due to its superior binding affinity and stability. Fortunately, ProMyc also plays the role of bridged PROTAC which not only significantly degrades c‐Myc, but also reduces Max protein, synergistically impairing c‐Myc‐driven gene expression, thus, providing a potential and multifunctional degrader.

To improve the pharmaceutical potential, we designed circPA1‐ProMyc which potently suppresses tumor growth in tumor xenograft mice, laying a solid foundation for the development of aptamer‐based degrader for the clinic. Moreover, circPA1‐ProMyc can simultaneous degradation of c‐Myc and PD‐L1. The c‐Myc oncogene also enables tumor immune evasion.^[^ ^26^ ^]^ We predict that the circPA1‐ProMyc would show a synergistic potential in immunotherapy. Additionally, this aptamer‐based degrader for proteolysis of the difficult‐to‐drug targets (ProDita) is also programmable by replacing the corresponding aptamer. We look forward to the modular ProDita for the degradation of the difficult‐to‐drug targets opening up an avenue for drug discovery and would be a promising therapy for incurable disease treatment.

## Conflict of Interest

The authors declare no conflict of interest.

## Supporting information

Supporting Information

## Data Availability

The data that support the findings of this study are available from the corresponding author upon reasonable request.
