# Supplementary material for: Antitumor Effect of Anti‐c‐Myc Aptamer‐Based PROTAC for Degradation of the c‐Myc Protein
Source: Adv Sci (Weinh). 2024 Apr 29;11(26):2309639. doi: 10.1002/advs.202309639 (PMC11234457; doi:10.1002/advs.202309639)
Supplement: Supplementary file 1 — Supporting Information [file ADVS-11-2309639-s001.pdf]

ADVANCED SCIENCE
Open Access

Supporting Information

for *Adv. Sci.*, DOI 10.1002/advs.202309639

Antitumor Effect of Anti-c-Myc Aptamer-Based PROTAC for Degradation of the c-Myc Protein

Yuchun Wang, Gang Yang, Xinyu Zhang, Ruoling Bai, Deyu Yuan, Denghui Gao, Qianyu He, Yi Yuan, Xinghe Zhang, Junchuang Kou, Lihua Zheng, Yanxin Huang, Zhuo Tang, Yongli Bao, Xu Song and Yongyun Zhao**

Supporting Information

Antitumor effect of anti-c-Myc Aptamer-based PROTAC for Degradation of the c-Myc protein

Yuchun Wang, Gang Yang, Xinyu Zhang, Ruoling Bai, Deyu Yuan, Denghui Gao, Qianyu He, Yi Yuan, Xinghe Zhang, Junchuang Kou, Lihua Zheng, Yanxin Huang, Zhuo Tang, Yongli Bao, Xu Song, Yongyun Zhao**

Experimental Procedures

Materials

Recombinant human c-Myc protein (Active) (ab169901), Recombinant Human MAX protein with His tag C-Terminus (ab95309), Anti-c-Myc antibody (ab32072), FITC Anti-c-Myc antibody (ab223913), Anti-CRBN antibody (ab226782), Anti-Ubiqutin antibody (ab134953), Goat Anti-Rabbit IgG H&L (Alexa Fluor® 594) (ab150080) were purchased from Abcam (UK). Anti-N-Myc antibody (10159-2-AP) was purchased from Proteintech (Rosemont, USA). Anti-L-Myc antibody (ER61985) was purchased from HuaBio (Hangzhou, China). Goat Anti-Rabbit secondary antibody, Goat Anti-Mouse secondary antibody were purchased from Thermo Fisher (A11008, A11001, USA). Pomalidomide-PEG4-C-COOH, VH032-PEG5-COOH were purchased from MCE (HY-21930, HY-130271, Shanghai, China). Protein G

beads were purchased from Thermo Fisher (91216610, USA). HRP-conjugated Beta Actin Monoclonal antibody was purchased from Proteintech Inc. (HRP-60008, USA). His-tag protein pure Ni-Beads (Ni-Beads) were purchased from BioMag Beads (BMNI-5, Wuxi, China). Exonuclease Ⅰ, was purchased from Sangon Biotech (C610019, Shanghai, China). Dip and Read™ Biosensors streptavidin (SA) were purchased from ForteBio (California, USA). The c-Myc inhibitor (MYCi361) (T12132) was purchased from TargetMol (Shanghai, China). The siRNA for knockdown CRBN was purchased from Yaoyuan (Shanghai, China). QuickEasy™ Cell Direct RT-qPCR Kit SYBR Green I (DRT-01012) was purchased from Forgene (Chengdu, China). Fast Silver Stain Kit (P0017S) was purchased from Byotime (Shanghai, China). Mouse Tumor Necrosis Factor α ELISA kit and Mouse Interleukin-6 ELISA kit were obtained from Meimian (MM 0122M1/MM-0163M1, Jiangsu, China). All media for cell culture were purchased from Gibco (USA). Fetal bovine serum (FBS) was purchased from Excell Bio (Shanghai, China) and penicillin-streptomycin was purchased from Hyclone (USA). All types of DNA sequences with HPLC purification were synthesized by Sangon Biotech (Shanghai, China).

Microwell-SELEX procedures

The recombinant human c-Myc protein without tags was used as the target and total protein in HCT116 cells with silencing c-Myc was used as the negative control. An ssDNA library (5’-CGTTACTTCTGTTCGTTCTCATG-[N]40- CCTAGTTCCTTGTCTGATCTCCA -3’ (N = A, T, G, or C)) consisting of 40 nt random core sequence and constant arm sequences at both ends were used for DNA library. Briefly, the untagged c-Myc protein (1 µg) was coated on a polystyrene microwell plate at 4 °C overnight. The synthetic ssDNA library (33 µg) was incubated with c-Myc proteins for 1 h at RT in binding buffer containing 1×PBS buffer with magnesium ions (10 mM Na_2HPO4, 2 mM KH_2PO4, 137 mM NaCl, 2.7 mM KCl, 0.55 mM $MgCl_2$, pH=7.4) and then separated and washed twice with the washing buffer (PBST: the binding buffer with 0.05 % Tween 20). Subsequently, the bound

oligonucleotides were eluted by Milli-Q H2O at 95℃ for 10 min with mild shaking. The selected ssDNA was amplified by PCR. To convert dsDNA to ssDNA, the Lambda exonuclease enzyme was applied to separate and remove the 5′-phosphorylated antisense sequences from the sense sequences. Then, the ssDNA purified using NucleoSpin® Extract II kit was subjected to the next round of SELEX.

After 3 rounds, the enriched ssDNA sequences (500 ng) were first incubated in control proteins for 30 min for counter-selection and the unbound ssDNA was then transferred to incubated with c-Myc. Additionally, to increase the selection pressure, after 3 rounds, the amount of c-Myc was decreased gradually from 1 μg to 0.5 μg. After 5 rounds, the concentration of Tween 20 increased gradually from 0.05 % to 2 %. The incubation time of ssDNA with c-Myc was decreased gradually from 1 h to 0.5 h. After 12 rounds of selection, the enriched libraries were subjected to high-throughput sequencing (Sangon Biotech, Shanghai, China).

Binding assays of the aptamer to protein

c-Myc proteins (100 ng per well) were coated in the 96-well ELISA Microplates with 100 μL coating buffer at 4 °C overnight. The c-Myc -protein-coated plates were washed twice with washing buffer before blocking with 200 μL of the solution (2% BSA in PBST) at RT for 1 h. Then, 200 nM final concentration biotin-labeled aptamer-ASO chimera at the 5′-end were added into the wells and incubated in binding buffer at RT for 30 min with gentle shaking. After washing two times, streptavidin-horseradish peroxidase (HRP) and its substrates were sequentially added to the reactions. Color development was carried out using 3,3′,5,5′- Tetramethylbenzidine (TMB) substrate and measured using a microplate reader at 450 nm absorbance.

Bio-layer interferometry (BLI)

BLI was carried out according to our previous research[1]. Briefly, the sensors were immersed into 96-well added 200 **μL** PBST/Mg^{2+} (0.55 mM) and shaking (1000 rpm) for 90 s (baseline phase). For loading purposes, streptavidin biosensors were used to capture biotinylated DNA

(the thickness signal was 0.6 nm) in PBST/Mg with shaking for 10 min (loading phase). After washing in the PBST/Mg^{2+} for 60 s (second baseline phase), the loaded sensors were immersed into a serial dilution of c-Myc proteins with shaking for 3 min (association phase). Then, the sensors were immersed into PBST/Mg^{2+} for an additional 3 min (disassociation phase). Binding affinity kinetic features using ForteBio Octet K2 (Pall, USA).

Flow cytometry analysis

To evaluate the binding performance of His-tagged c-Myc proteins (50 nM) coated in Ni-beads were incubated with 200 nM cy3-labeled aptamer-ASO chimera in 100 µL binding buffer at RT for 30 min. Meanwhile, Ni-beads incubated with cy3-labeled random sequences were used as the negative control. The beads were washed twice using washing buffer and suspended in 1 mL binding buffer. The fluorescence intensity of beads with counting about 5000 events was measured by flow cytometry (FACSVerse, BD). Meanwhile, the data was analyzed using FlowJo (V10, BD).

Cell culture

HCT116, A549, MDA-MB-231, HeLa, and HEK-293T cells were obtained from the Shanghai Institute of Cell Biology cell bank (Chinese Academy of Medical Sciences, Shanghai, China). HCT116, MDA-MB-231, A549, and HEK-293T cells were cultured in DMEM medium (Gibco) supplemented with 100 U/mL Penicillin-Streptomycin, 2 µg/mL Puromycin and 10 % (v/v) fetal bovine serum (ExCell Bio). HeLa cells were cultured in MEM medium (Gibco) supplemented with 100 U/mL Penicillin-Streptomycin and 10 % (v/v) fetal bovine serum (ExCell Bio). All cell lines were cultured in a humidified atmosphere containing 5% CO_2 incubator at 37 °C.

Streptavidin-Biotin pull-down Assay

HTK-293T cells expressing His-c-MYC were lysed ultrasonication in the PBS buffer including 0.2% Tween 20 and 0.1% PMSF 0.55mM $MgCl_2$. The protein concentrations of the lysates were measured

using the Bio-Rad protein assay reagent on a Beckman Coulter DU-800 spectrophotometer. The cell lysates (1 mg) were further incubated with biotin-aptamer (10 µg) at RT for 2 h, followed by the addition of 20 µL of Streptavidin agarose beads (Thermo Fisher) for another 1 h. The beads were washed 5 times with wash buffer1 (100 mM NaCl, 20 mM Tris-Cl, pH 8.0, 0.5 mM EDTA, 0.5% NP-40), boiled in SDS loading buffer, further separated by 10% SDS-PAGE, and blotted with individual antibody.

Mass Spectrometry Analysis

The mass spectrometry analysis was performed as previously reported[2]. The modification of biotin at the 5-terminal of MA9C1 reduces the binding efficiency, here, we used the MA9 sequence. HCT116 cells were lysed in 5 mL of hypotonic buffer (50 mM Tris-HCl containing protease inhibitors) at 4 °C for 30 min. After centrifugation, the debris was washed three times with 5 mL of hypotonic buffer and dissolved in 1.5 mL of lysis buffer (PBS containing 5 mM $MgCl_2$ and 1% Triton X- 100) at 4 °C for 30 min. The supernatant was incubated with beads, mutMA9, or MA9 at 4 °C for 1 h, respectively. Subsequently, the protein-DNA complex was captured by incubating it with 2 mg (200 µL) of streptavidin beads for 45 min at 4 °C. Then, the proteins were eluted by heating in 30 µL of loading buffer and analyzed by SDS-PAGE gel and Silver staining. The differential strand and aptamer-purified protein bands were excised for digestion in situ and analyzed by LC-MS/MS at the Shanghai Primerna NAT Co. Ltd. (shanghai, China).

Molecular Docking Methods

The protein structure (c-Myc_human) was accurately predicted using the AlphaFold database. The secondary structure of the adaptor was reliably forecasted using the mfold web server, and the tertiary structure was inferred using the 3dDNA tool. Docking simulations were carried out using the locally installed Hdocklite software (version 1.1) with Grid spacing and Angle step parameters set to 1.200 and 15.000, respectively. Among the various predicted docking conformations, the model with the lowest binding score (model 1, Docking Score: -456.34) was selected for further analysis. The docking

score was calculated using the knowledge-based iterative scoring functions ITScorePP or ITScorePR. Finally, a comprehensive interaction analysis was performed using the PDBePISA server.

Pomalidomide-DNA conjugate preparation

0.2 M Phosphate Buffer (PB buffer, Na_2HPO_4, NaH_2PO_4, PH=8.0) was used to solubilize the 5'-aminomodified DNA oligonucleotide MA9C1, and the final concentration was 100 µM. 1 mg carboxyl-labeled pomalidomide derivative was first activated in 200 µL activation buffer (0.1 M MES (2-[morpholino]ethanesulfonic acid), 0.5 M NaCl, pH 6.0) containing 15 mM DCC (Dicyclohexylcarbodiimide) and 15 mM NHS (NHydroxysulfosuccinimide sodium salt) by shaking at 100 rpm at 25°C for 30 min. Mix DNA and pomalidomide. The molar ratio of DNA to pomalidomide was 1:20. The reaction was carried out under gentle stirring of 700 rpm at 25°C for overnight. The product (pomalidomide-DNA conjugate, ProMyc) was collected by ethanol precipitation and purified by Ultra-High Performance Liquid Chromatography (UPLC).

Luciferase reporter gene assay

HCT116 cells were seeded in 12-well plates at 1×10^6 cells/well. The cells were co-transfected with c-MYC luciferase reporter plasmid (Tbo, YB046B) and oligonucleotide (50 nM) using Lipofectamine 2000 according to the manufacturer's protocol. After 48 hours, cells were used for measuring luciferase activity according to the ONE-Glo™ Luciferase Assay System (Promega, TM292).

Cell cycle distribution

The cell cycle distribution was profiled using propidium iodide (PI) staining followed by fluorescence-activated cell sorting (FACS) analysis. In brief, cells were trypsinized into single-cell suspension, rinsed three times with ice-cold PBS and fixed in ice-cold 70% ethanol for 2 h. To remove the effects of RNA, the cells were treated with 100 g/ml RNase A at 37 °C for 30 min before being stained with PI (50 µg/ml) at 4 °C for 30 min. The cells were subjected to FACS analysis using the BD cytometer (BD Biosciences). The data was analyzed using FlowJo software (Treestar).

Immunofluorescence

The cells were seeded in culture dishes and incubated for 12 h. Cells were treated with MG132 (20 μM) for 1 h, followed by transfected with ProMyc (50 nM) for 24 h. Then the cells were fixed with 4% paraformaldehyde in the dark at room temperature for 30 min, and permeabilized with 0.1% Triton-X 100 for 10 min. After blocking with 1% BSA for 1 h, the cells were incubated with anti-c-Myc primary antibody (1:100, Y69, ab32072) at RT for 1 h. Then, the cells were washed with PBST (156 mM NaCl, 3 mM Na_2HPO4, 1 mM KH_2PO4, 0.1% Tween-20, pH 7.4) twice, stained with Alex 555-labeled secondary antibody (1:500, ab150078) at RT for 1 h, and then washed twice. Finally, the cells were stained with Hoechst 33258 at RT for 10 min, and washed with PBST for twice. The cell photographs were acquired with an Olympus Laser Scanning Confocal Microscope.

Co-immunoprecipitation assay (Co-IP)

HEK-293T cells were firstly transfected with His-c-MYC (12 μg) and Flag-CRBN (12 μg) plasmid for 24 h, followed by additional treatment with MG132 (20 μM) for 1 h. The cells were secondly transfected with ProMyc (200 nM) for another 12 h. After being harvested, cells were lysed with lysis buffer (20 mM Tris-HCl [pH 8.0], 100 mM NaCl,1 mM EDTA, and 0.5% Nonidet P-40). The lysates were then incubated at RT for 2 h. 1 mg of protein from the lysate was incubated with Anti-FLAG Magnetic Beads (Sigma) at RT for 1 h. Samples then were washed 3 times with lysis buffer. 3× FLAG Peptide (Sigma) was used to release the binding proteins. The eluted fractions were analyzed by Western blotting.

In vivo ubiquitination assay

HEK-293T cells were firstly transfected with HA-ubiquitin plasmid (12 μg) for 24 h, followed by additional treatment with MG132 (20 μM) for 1 h. The cells were secondly transfected with ProMyc (50 nM) for another 12 h. After being harvested, cells were lysed, and lysates were then incubated with anti-Myc antibody at 4 °C overnight followed by protein G agarose beads for an additional 1 h at

room temperature. The proteins were released from beads by boiling the beads in an SDS-PAGE sample buffer. The ubiquitinated c-Myc were finally separated by 7% SDS-PAGE and blotted with anti-Ub or anti-HA antibodies.

Western blot analysis

Cells were washed twice with PBS and dissolved in lysis buffer (50 mM Tris-HCl pH 7.4, 300 mM NaCl, 1% Triton X-100, 5 mM EDTA, 100 mM phenylmethylsulfonyl fluoride (PMSF) and 10% glycerol) after transfection. Protein concentration was measured by Bicinchoninic Acid assay (BCA). Proteins were separated by SDS polyacrylamide gel electrophoresis (SDS-PAGE), transferred onto PVDF membranes (Bio-Rad) and incubated overnight at 4 ℃ with corresponding antibodies: anti-c-Myc (1:20000), anti-ACTB-HRP (1:10000). A HRP conjugated Rabbit anti-mouse IgG secondary antibody (1:10000) was used for the detection of c-Myc. The protein signals were detected using an ECL chemiluminescent substrate (FOREGENE, China).

Agarose gel electrophoresis analysis of aptamer stability

Oligonucleotide (500 ng) was added to the binding buffer containing 10 % FBS and incubated at 37 °C. Then, the samples were loaded onto 2 % agarose gel in 1×TAE buffer and run at 120 V for 30 min. Gels were incubated in Ethidium bromide (1 μg/ml) for 5 min and bands were imaged using Gel Doc XR + Gel imaging system (Bio-rad, Hercules, California, USA) with ultraviolet excitation (302 nm).

Competition ELISA assay

Briefly, 96-well ELISA Microplates were pre-coated with c-Myc proteins in 100 μL coating buffer (pH=9.6, 100 mM $NaHCO_3$) at 4 ℃ overnight. 200 μL blocking solution was added at room temperature (RT) for 1 h with mild shaking. Serial dilutions of biotin-labeled oligonucleotide (100 nM) and unlabeled oligonucleotide (200 nM) were added into microplates and incubated at RT for 30 min. Then, HRP-conjugated streptavidin and TMB substrate were used to measure the absorbance at 450 nm on a microplate reader.

Quantitative RT-PCR (qRT-PCR) analysis

Total RNA was isolated from the stimulated cells by RNAiso Plus (108-95-2, TaKaRa, Japan). Then, cDNA was prepared using DNase I (01056834, Thermo Scientific, USA) and RevertAid Reverse Transcriptase (00991337, Thermo Scientific, USA). qRT-PCR was performed using the Applied Biosystems 7500 Real-Time PCR Systems (Thermo Fisher Scientific, USA) with 2×Real PCR Easy™ Mix-SYBR (210520, Foregene, China). The data of qRT-PCR were analyzed by the Livak method (2-ΔΔCt). ACTB was used as a reference gene for the human cell lines.

Construction of circular chimera

The preparation of circular chimera was carried out according to our previous research[3]. PA1-MA9C1 was modified phosphate group at 5'- terminus to combine with 3'-OH. Subsequently, PA1-MA9C1 was dissolved in T4 DNA Ligase buffer and heated at 95°C for 5 min, followed by rapid chilling to 4 °C. Then, T4 DNA ligase (NEB, America) was quickly added to the solution and reacted at 16 °C for 12 h. Afterward, the T4 DNA ligase was denatured at 65°C for 10 min, according to the manufacturer's instructions.

Confocal microscope imaging of HCT116 cells incubated with cicPA1-ProMyc

Confocal microscope imaging was carried out according to our previous research[4]. Briefly, HCT116 cells were incubated with cy3-labeled DNA at 37 °C for 24 h. After incubating with Hoechst 33342 for 15 min, HCT116 cells were harvested to image with a Zeiss CELL Observer SD confocal microscope with a 60 × oil objective. The excitation wavelengths were 488 nm (Green Channel) and 561 nm (Red Channel). Exposure times: 200–400 ms. Acquired images were analyzed by the ZEN (blue edition).

Cell proliferation and clonal formation assay

HCT116 cells were seeded at a density of 1×10^6 in 12-well plates. After 8-12 h cell culture, the cells were transfected with ProMyc or incubated with cicPA1-ProMyc. Then the cells were trypsined and the cell numbers were counted. Cells were seeded at a density of 1×10^3 in 96-well plates. After cell culture, cell viability was assessed using a CCK-8 kit (Beyotime, China). The absorbance at 450 nm was measured on a microplate reader. The mean value of the wells with media alone was used as background and was subtracted from the absorbances of the wells containing cells.

For the clonal formation assay, HCT116 cells were transfected with or incubated with cicPA1-ProMyc for 6 h, followed by being further plated into a 6-well plate (2000/well). One week later, the cells were fixed in fixation buffer (4% paraformaldehyde) and stained with 0.4% crystal violet in 20% ethanol. Then, the clonal numbers were quantified by ImageJ software.

Mice

Female BALB/c mice (4-5 weeks old) were purchased from Changchun GemPharmatech Co. Ltd or Chengdu GemPharmatech Co. Ltd. All the mice were kept in sterile, autoclaved cages and provided with enough food and water. All animal experiments were undertaken at Northeast Normal University and Sichuan University. All protocols were approved by the Institution. All experiments were approved by the local regulatory agency.

Tumor Xenograft Study

Nude Mice（female, 6-8 weeks old) were injected subcutaneously in the flank with HCT116 cells (3×10^6 cells) suspended in serum-free media. Mice were exposed via peritumoral injection with either PBS or 5 mg/kg every 4 days starting 7 days after injection of cancer cells. The tumor volumes and body weights were monitored every 2 days. Tumor size was assessed by caliper measurements. The formula: V (mm^3) = 1/2× length (mm) ×width $(mm)^2$ was used to calculate tumor volume (V). Each measurement was performed three times, and recorded the mean value as the final tumor volume. Tumors were dissected and photographed at 19 days post-inoculation.

ProMyc induces cytokine responses in mice

Female BALB/c mice (4-5 weeks old) were provided by Changsheng Biotechnology Co Ltd. (Liaoning, China). For in vivo stimulation, 200 μL physiological saline solution containing DNA was incubated for 20 mins. Subsequently, mice (BALB/c) were randomly separated into 3 groups and injected through intravenous injection. The administration amount of the ProMyc is 10 mg/kg. Control mice received either physiological saline solution (blank) alone or LPS (3 mg/kg) (positive control), respectively. Whole blood samples were obtained by tail clippings at the indicated time and serum was prepared from whole blood by coagulation at 37 °C for 30 min and centrifugation. Mouse cytokine (TNF-α, IL-6) was measured using a mouse cytokine ELISA kit according to the product information and manual.

Statistical analysis

All analyses were repeated at least three times, and a representative experimental result was presented. Data were analyzed using GraphPad Prism version 8.0 (GraphPad Software, San Diego, CA). Continuous variables with normal distribution are expressed as the mean ± standard deviation (SD). Student's t-test (for pairwise comparisons) and one- or two-way ANOVA (for comparisons among three or more groups) were used. Statistical and quantification details of experiments can be found in the Figure legends.

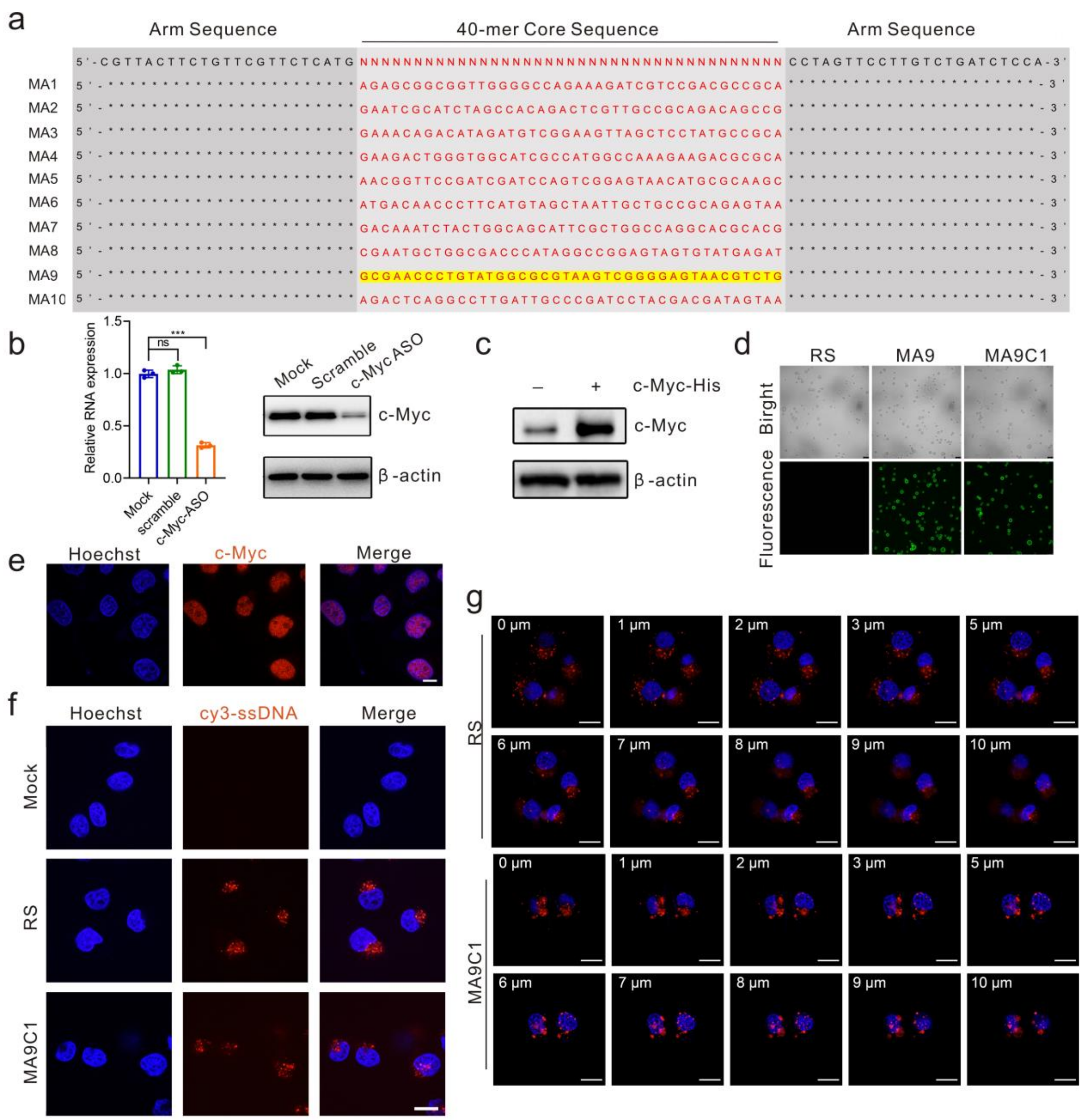

Supplementary Figure S1. Identification and characteristics of aptamer MA9C1. a. The sequences of 10 candidate sequences. Arrows show 23-mer consistent arm sequences at both ends for primer annealing of PCR amplification. Aptamer MA9 was highlighted in yellow. **b.** qRT-PCR and representative western blot analysis of c-Myc in HCT116 cells transfected with c-Myc ASO (150 nM) for 48 h. Mock: without oligonucleotides. Scramble: a nontargeting control ASO. **c**. Purified His-tagged c-Myc in HEK-293T cells transfected with plasmid expressing His-tagged c-Myc. **d**. Fluorescence images to detect the binding performance of FAM-labeled candidate sequences (200 nM) to 100 µL binding buffer

containing His-c-Myc-beads (5 µl beads and 2 µg His-c-Myc). Rs: the random sequence. **e**. Immunofluorescence images of c-Myc in HCT116 cells. Red signals indicate c-Myc. The nucleus is stained with Hoechst 33258. Scale bar, 10 µm. **f**. Representative fluorescence images of A549 cells transfected with 100 nM cy3-labeled aptamer MA9C1. The nucleus is stained with Hoechst 33258. Scale bar, 10 µm. **g**. 3D projections of Z-stack images of A549 cells transfected with 100 nM cy3-labeled aptamer MA9C1 at different relative focus positions. Scale bar, 10 µm.

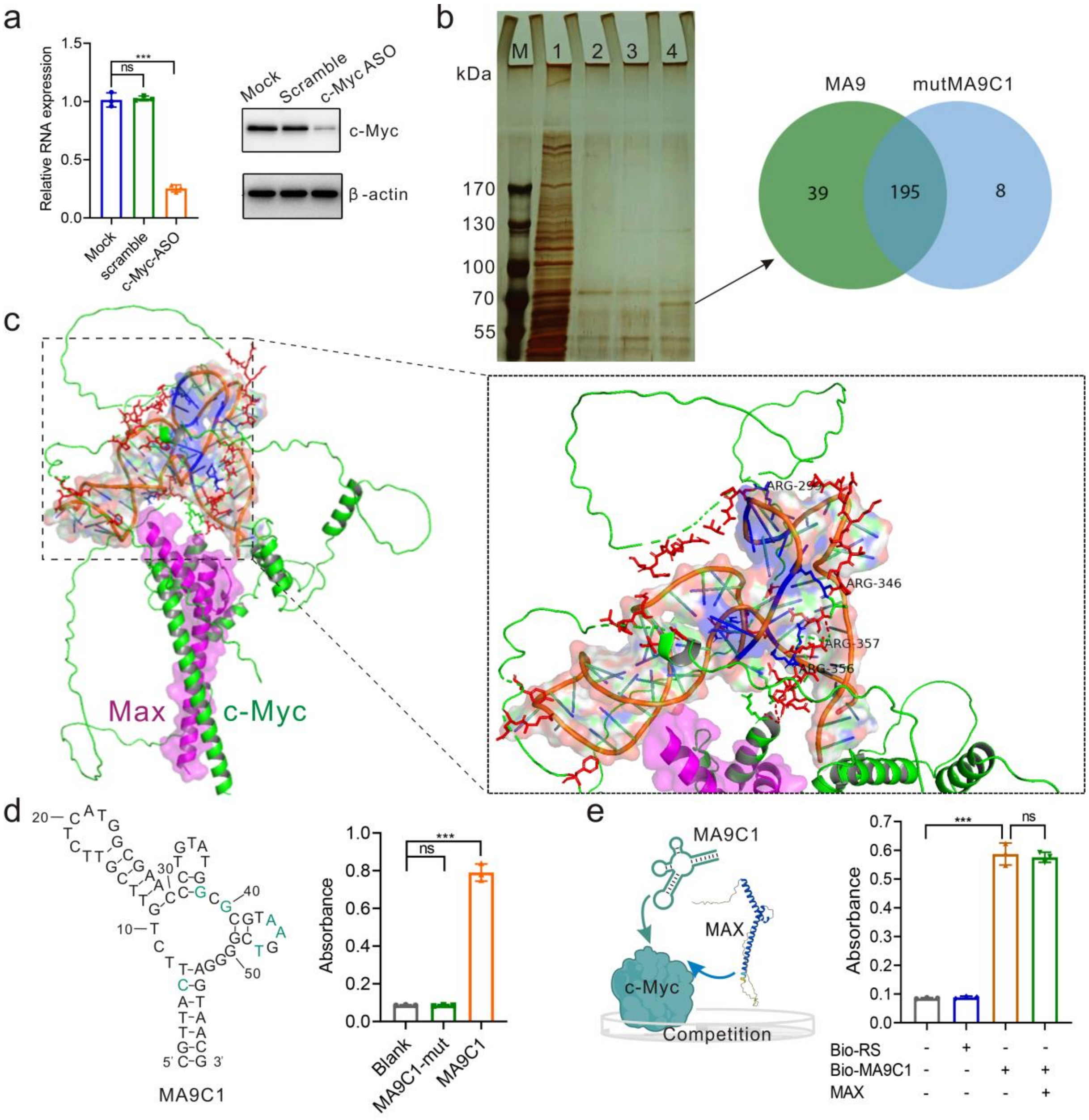

Supplementary Figure S2. The specificity and binding site of aptamer. a. qRT-PCR and representative western blot analysis of c-Myc in A549 cells transfected with c-Myc ASO (150 nM) for 48 h. Mock: without oligonucleotides. Scramble: a nontargeting control ASO. **b.** Silver-stained SDS-PAGE and LC-MS analysis was used to analyze the specificity of aptamer. Total proteins of HCT116 cells were incubated with biotin-labeled MA9 or mutMA9C1, respectively. M: molecular markers; Lane 1: the input; Lane 2: magnetic beads only; Lane 3: protein captured with mutMA9C1; 4: protein captured with the MA9. LC-MS analysis of the distinguishing bands. **c**. Interaction model between c-Myc and MA9C1 and close view of c-Myc and MA9C1 interaction region in part. The interfacing residues are marked in red. The key hydrogen bonds are marked in blue as follows: C6/ARG357, G38/ARG356, G40/ARG346, A44/ARg299, A45/ARg299, T47/ARg299. **d**. The key bases for forming hydrogen bonds were mutated to verify the correctness of molecular docking by ELISA. The bases labeled green have been artificially mutated (MA9C1-mut). **e.** Competition ELISA to evaluate the ability of aptamer MA9C1 (200 nM) that prevents c-Myc (200 ng/well) binding to MAX (100 ng/well) pre-coated on microplates. The random sequence (RS) used as the negative control is the ssDNA initiation library. All the error bars indicate standard deviations (n= 3). All the *P* values were determined. ***$P < 0.001$.

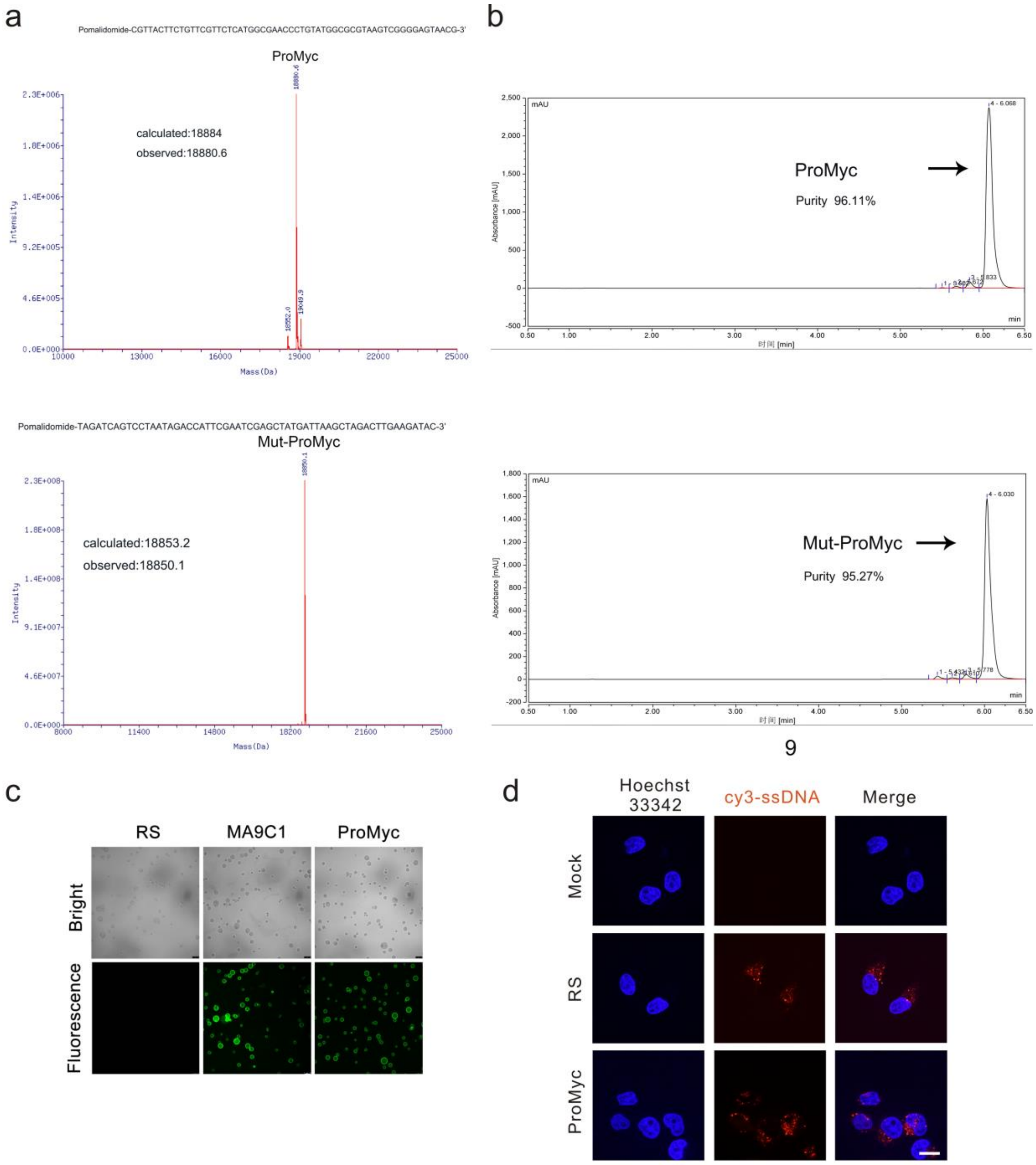

Supplementary Figure S3. The synthesis and characteristics of ProMyc. a. Electrospray ionization mass spectrometry spectra of aminomodified ssDNA and ssDNA-pomalidomide conjugate. **b.** Purification of pomalidomide-linked DNA strand by UPLC. Free DNA was eluted first and DNA-pomalidomide conjugate was eluted later. **c**. Fluorescence images to monitor the binding performance of FAM-labeled ProMyc (200 nM) to 100 μL binding buffer containing His-c-Myc-beads (5 μl beads and 2 μg His-c-Myc). Rs: the random sequence. **d**. Representative

fluorescence images of A549 cells transfected with 100 nM cy3-labeled ProMyc. RS: the random sequence. The nucleus is stained with Hoechst 33258. Scale bar, 10 μm.

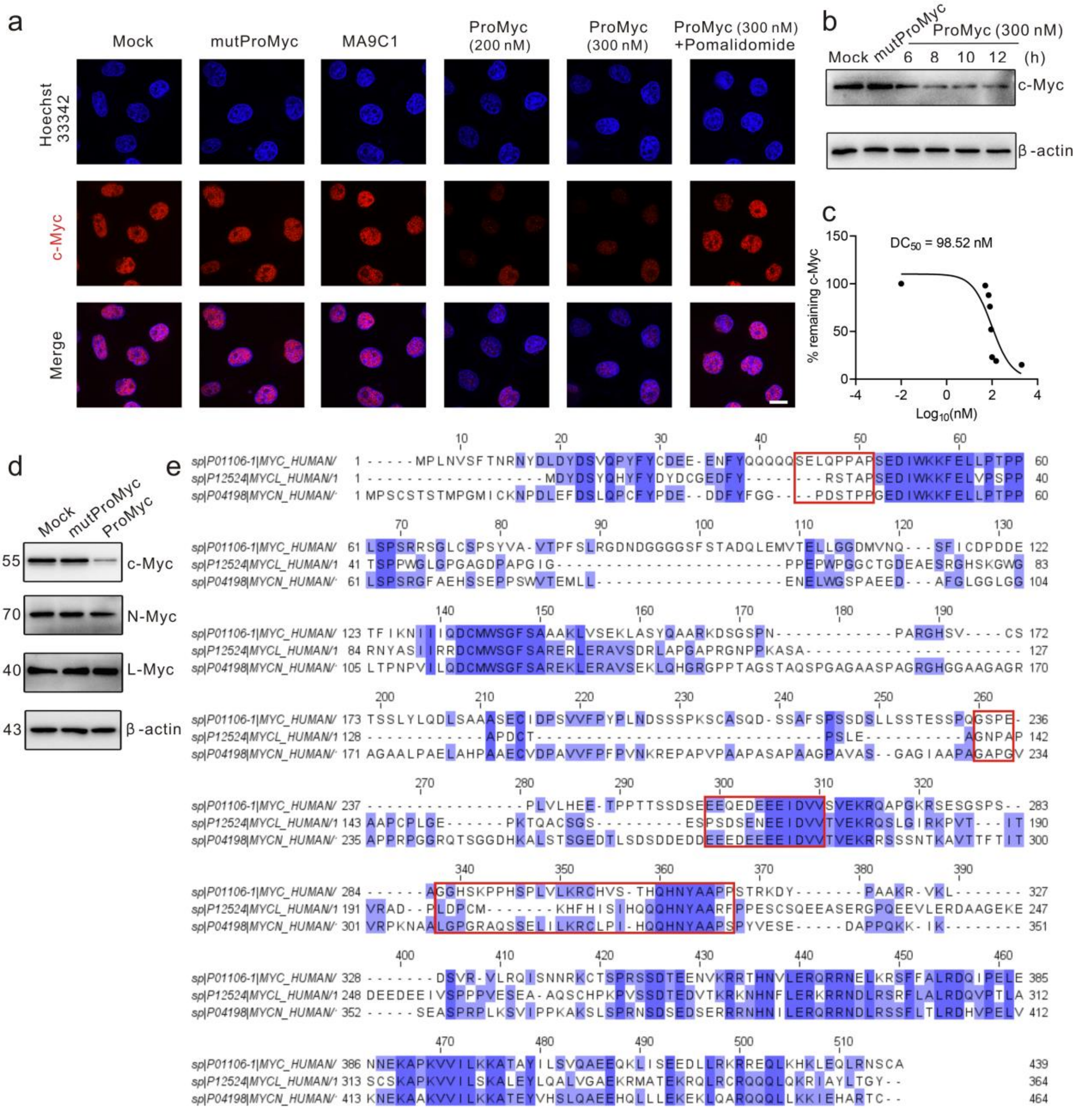

Supplementary Figure S4. ProMyc effectively degrades the endogenous c-Myc in A549 cells. a. Representative immunofluorescence images of c-Myc in A549 cells after 24 h treatment of ProMyc with the indicated concentration (or not) followed by Pomalidomide treatment. Red signals indicate c-Myc. The nucleus is stained with Hoechst 33258. Scale bar,

20 μm. **b**. The remaining c-Myc (%) was calculated by normalizing the value in each group to that in the Mock in Fig. 3e. And DC_{50} was determined. **c**. Western blot analysis of c-Myc protein in A549 cells transfected with ProMyc (300 nM) at the indicated time. **d**. The effect of ProMyc on the c-Myc homologous proteins (N-Myc and L-Myc). **e**. Bioinformatics analysis of the differences between c-Myc homologous proteins. The red box is the major binding site between MA9C1 and c-Myc. Homologous regions are marked in blue.

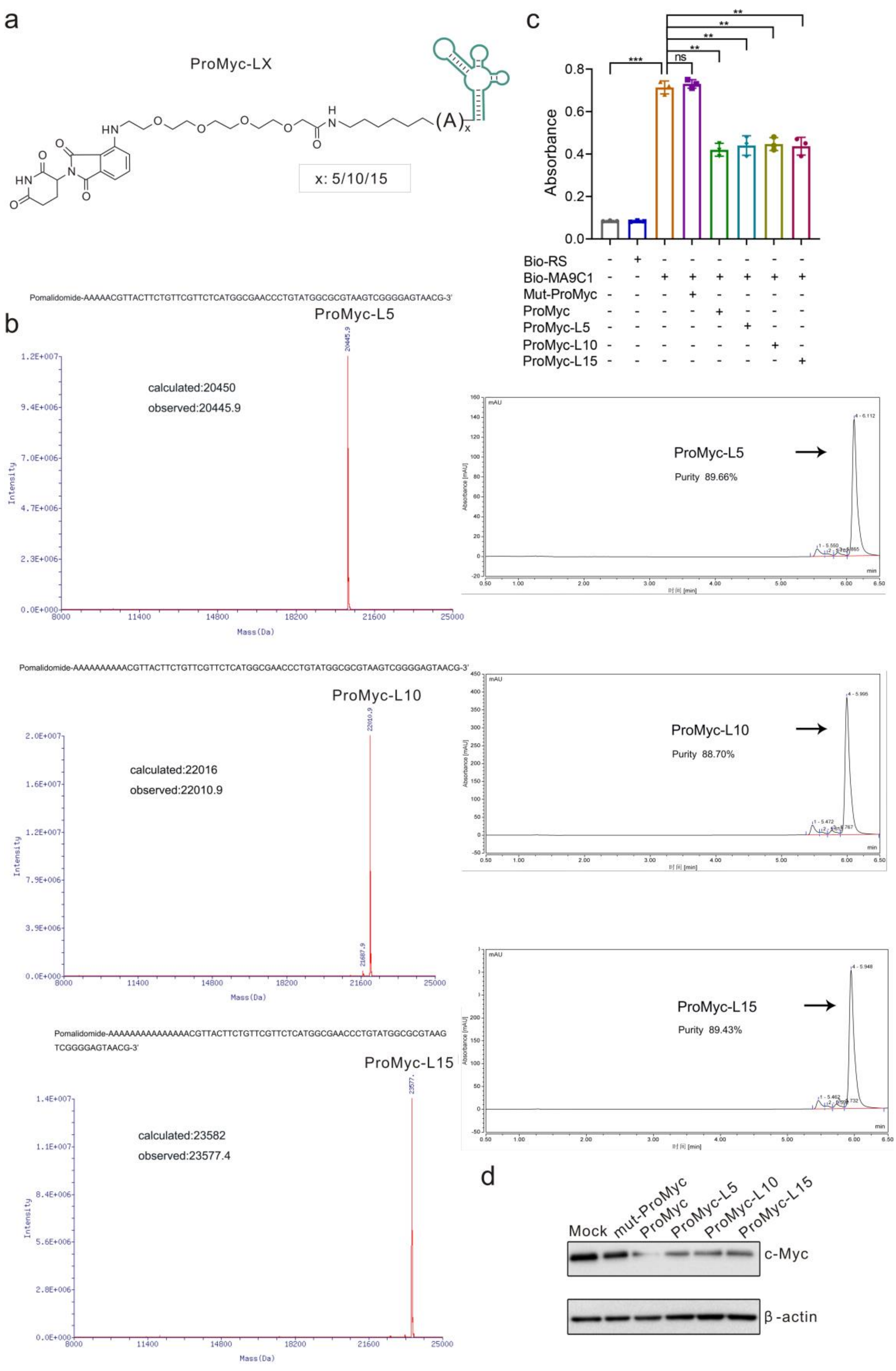
a
ProMyc-LX
x: 5/10/15
(A)x
c
Absorbance
Bio-RS
Bio-MA9C1
Mut-ProMyc
ProMyc
ProMyc-L5
ProMyc-L10
ProMyc-L15
ns
b
Pomalidomide-AAAAACGTTACTTCTGTTCGTTCTCATGGCGAACCCTGTATGGCGCGTAAGTCGGGGAGTAACG-3'
ProMyc-L5
calculated:20450
observed:20445.9
Intensity
Mass(Da)
ProMyc-L5
Purity 89.66%
Pomalidomide-AAAAAAAAAACGTTACTTCTGTTCGTTCTCATGGCGAACCCTGTATGGCGCGTAAGTCGGGGAGTAACG-3'
ProMyc-L10
calculated:22016
observed:22010.9
ProMyc-L10
Purity 88.70%
Pomalidomide-AAAAAAAAAAAAAAACGTTACTTCTGTTCGTTCTCATGGCGAACCCTGTATGGCGCGTAAG
TCGGGGAGTAACG-3'
ProMyc-L15
calculated:23582
observed:23577.4
ProMyc-L15
Purity 89.43%
d
Mock
mut-ProMyc
ProMyc
ProMyc-L5
ProMyc-L10
ProMyc-L15
c-Myc
β-actin

Supplementary Figure S5. The degradation efficiency of ProMyc with different linkers. **a.** The diagram of Pomalidomide-PEG4-C-COOH is linked to the C6-aptamer MA9C1 with different linkers via an amide bond. **b.** Electrospray ionization mass spectrometry spectra of amino-modified ssDNA and ssDNA- pomalidomide conjugate and their corresponding UPLC. **c.** Competition ELISA to evaluate the binding performance of those ProMyc-Lx (200 nM) to c-Myc. Biotin-labeled aptamer MA9C1 (200 nM) was mixed with different promyc-LX (200 nM) to bind to c-Myc (100 ng/well) pre-coated in a microplate. **d**. The representative western blot analysis of the c-Myc in HCT116 cells treated with different ProMyc-Lx (50 nM, L0, L5, L10, L15) for 24 h. All the error bars indicate standard deviations (n= 3). All the *P* values were determined. $^{**}P < 0.005$.

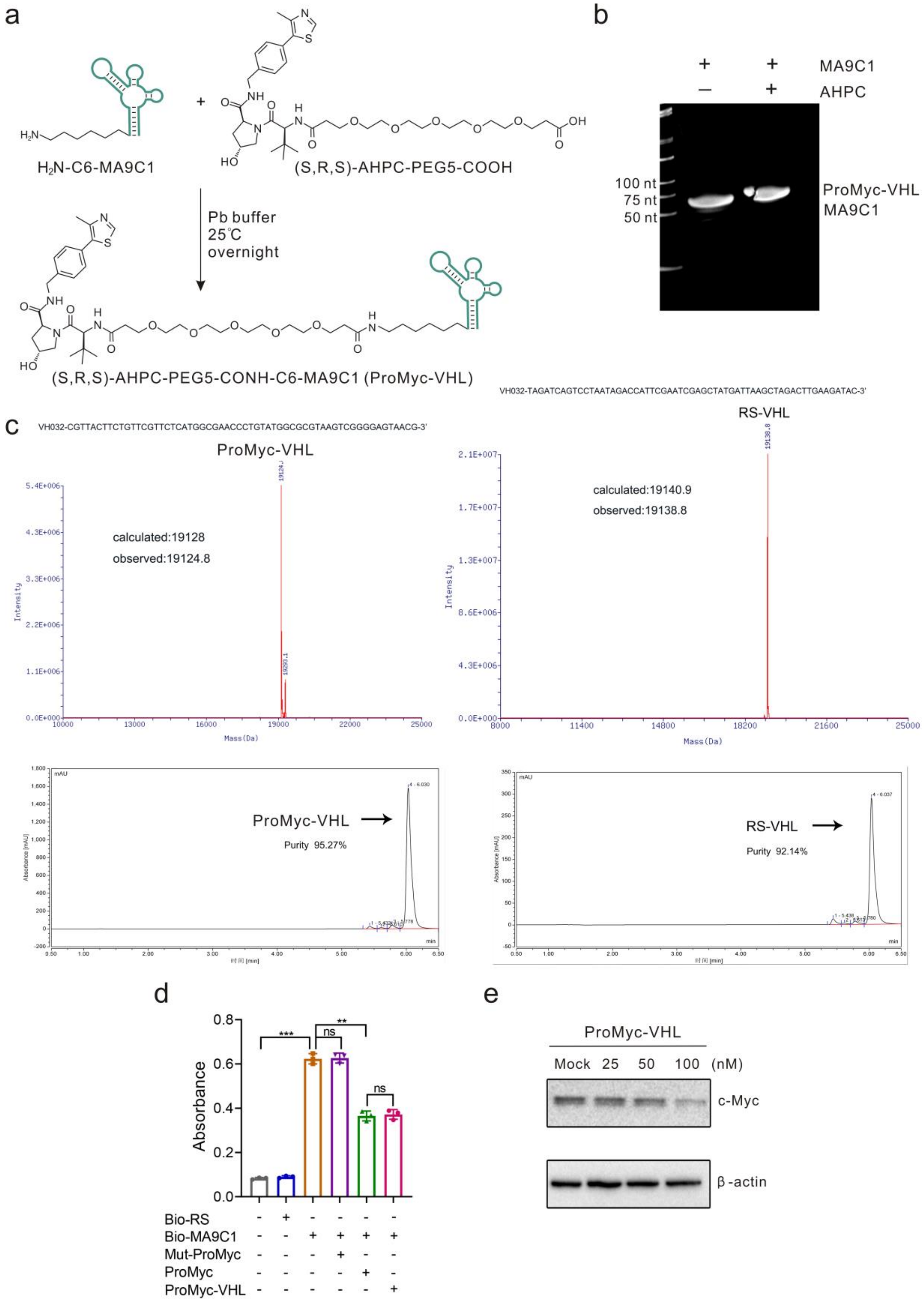
a
H2N-C6-MA9C1
(S,R,S)-AHPC-PEG5-COOH
Pb buffer
25℃
overnight
(S,R,S)-AHPC-PEG5-CONH-C6-MA9C1 (ProMyc-VHL)
b
MA9C1
AHPC
100 nt
75 nt
50 nt
ProMyc-VHL
MA9C1
c
VH032-CGTTACTTCTGTTCGTTCTCATGGCGAACCCTGTATGGCGCGTAAGTCGGGGAGTAACG-3'
ProMyc-VHL
calculated:19128
observed:19124.8
VH032-TAGATCAGTCCTAATAGACCATTCGAATCGAGCTATGATTAAGCTAGACTTGAAGATAC-3'
RS-VHL
calculated:19140.9
observed:19138.8
Intensity
Mass(Da)
ProMyc-VHL
Purity 95.27%
RS-VHL
Purity 92.14%
d
Absorbance
Bio-RS
Bio-MA9C1
Mut-ProMyc
ProMyc
ProMyc-VHL
e
ProMyc-VHL
Mock 25 50 100 (nM)
c-Myc
β-actin

Supplementary Figure S6. The degradation efficiency of ProMyc with VHL E3 ligase. a. The diagram of (S, R, S)-AHPC-PEG5-COOH is linked to the C6-aptamer MA9C1 via an amide bond to form ProMyc-VHL. **b.** 20% native PAGE analysis of the incorporation of AHPC onto the aptamer MA9C1. **c**. Electrospray ionization mass spectrometry spectra of amino-modified ssDNA and ssDNA-AHPC conjugate and their corresponding UPLC. **d**. Competition ELISA assay analysis of the binding performance of ProMyc-VHL to c-Myc. **e**. Representative western blot analysis of the c-Myc in HCT116 cells treated with different concentrations of ProMyc-VHL for 24 h. All the error bars indicate standard deviations (n= 3). All the *P* values were determined. ***P*＜0.005; ****P*＜0.001.

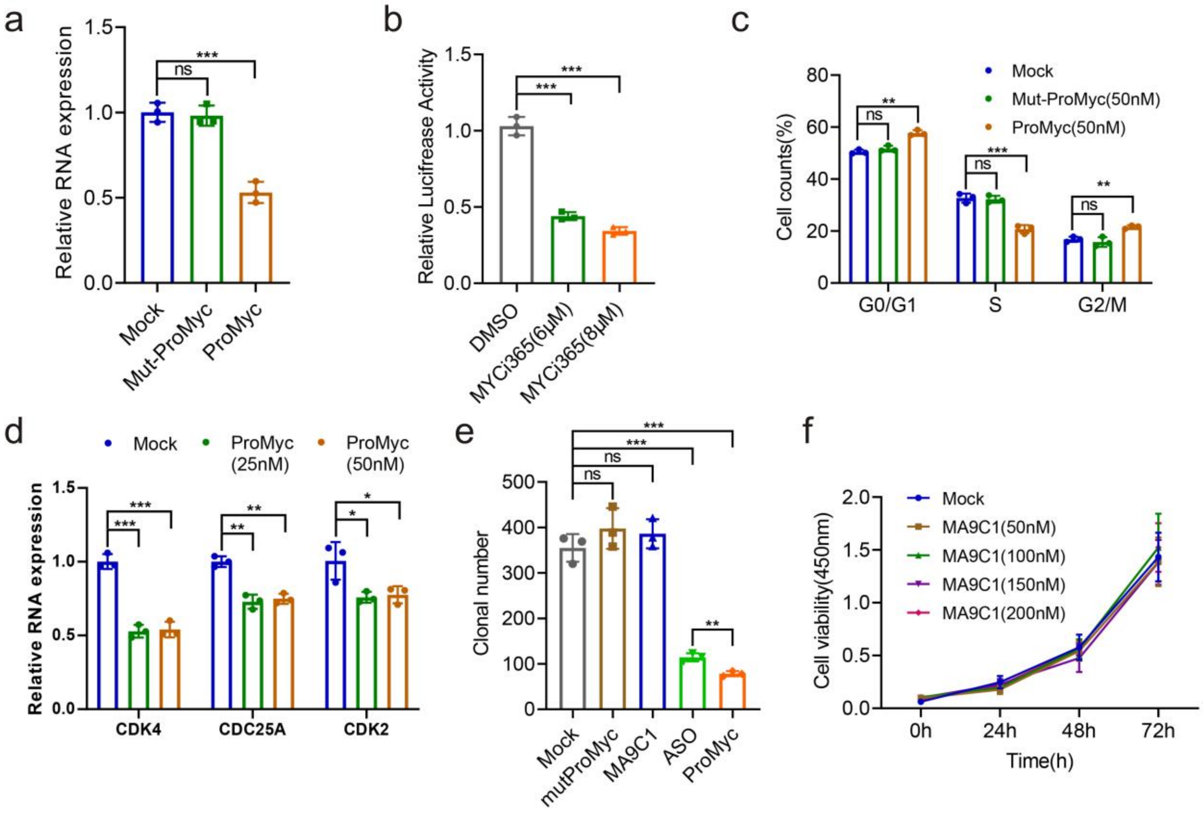

Supplementary Figure S7. a. qRT-PCR analysis of the luciferase gene expression in HCT116 cells transfected with different concentrations of ProMyc (50 nM) for 48 h. b.

HCT116 cells were treated with the c-Myc inhibitor (MYCi361) for 4 hr and luciferase activity was determined. The concentration of MYCi361 is 6 μM and 8 μM[5], respectively. **c**. FACS analysis revealed the cell cycle distribution of HCT116 cells with ProMyc (50 nM) treatment for 72 h. **d.** qRT-PCR analysis of the c-Myc- driven gene expression in HCT116 cells with ProMyc (50 nM) treatment for 48 h. **e.** Quantitative analysis of the inhibition of ProMyc on HCT116 cell migration and tumorigenicity in the colony formation assay. **f**. CCK8 assay analysis of the toxicity of aptamer MA9C1. All the error bars indicate standard deviations (n= 3). All the *P* values were determined. $^{**}P < 0.005$; $^{***}P < 0.001$.

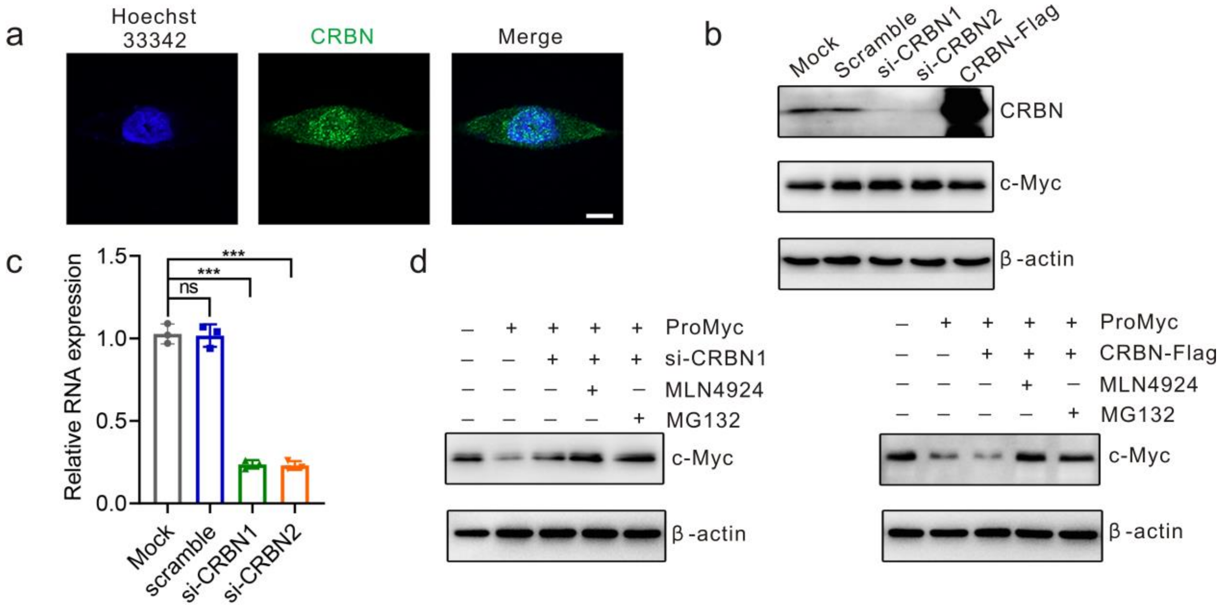

Supplementary Figure S8. a. Representative immunofluorescence images of the subcellular localization of CRBN with the anti-CRBN antibody. Green signals indicate CRBN E3 ligase. The nucleus is stained with Hoechst 33258. Scale bar, 10 μm. **b.** Representative western blot analysis of c-Myc protein in HCT116 cells transfected with si-CRBN (30 nM) or overexpress CRBN. Mock: without oligonucleotides. Scramble: a nontargeting control RNA. **c**. qRT-PCR analysis of c-Myc mRNA in HCT116 cells transfected with si-CRBN (30 nM) for 48 h. **d.** c-Myc protein levels in HCT116 cells treated with ProMyc in the presence of proteasome inhibitor MLN4924 or proteasome inhibitor MG132. The cells

were transfected with siCRBN (b) or plasmids expressing Flag-CRBN (c) for 48 h, and then incubated with MLN4924 (20 μM) or MG132(20 μM) for 1 h, followed by treatment with ProMyc (50 nM) for 24 h.

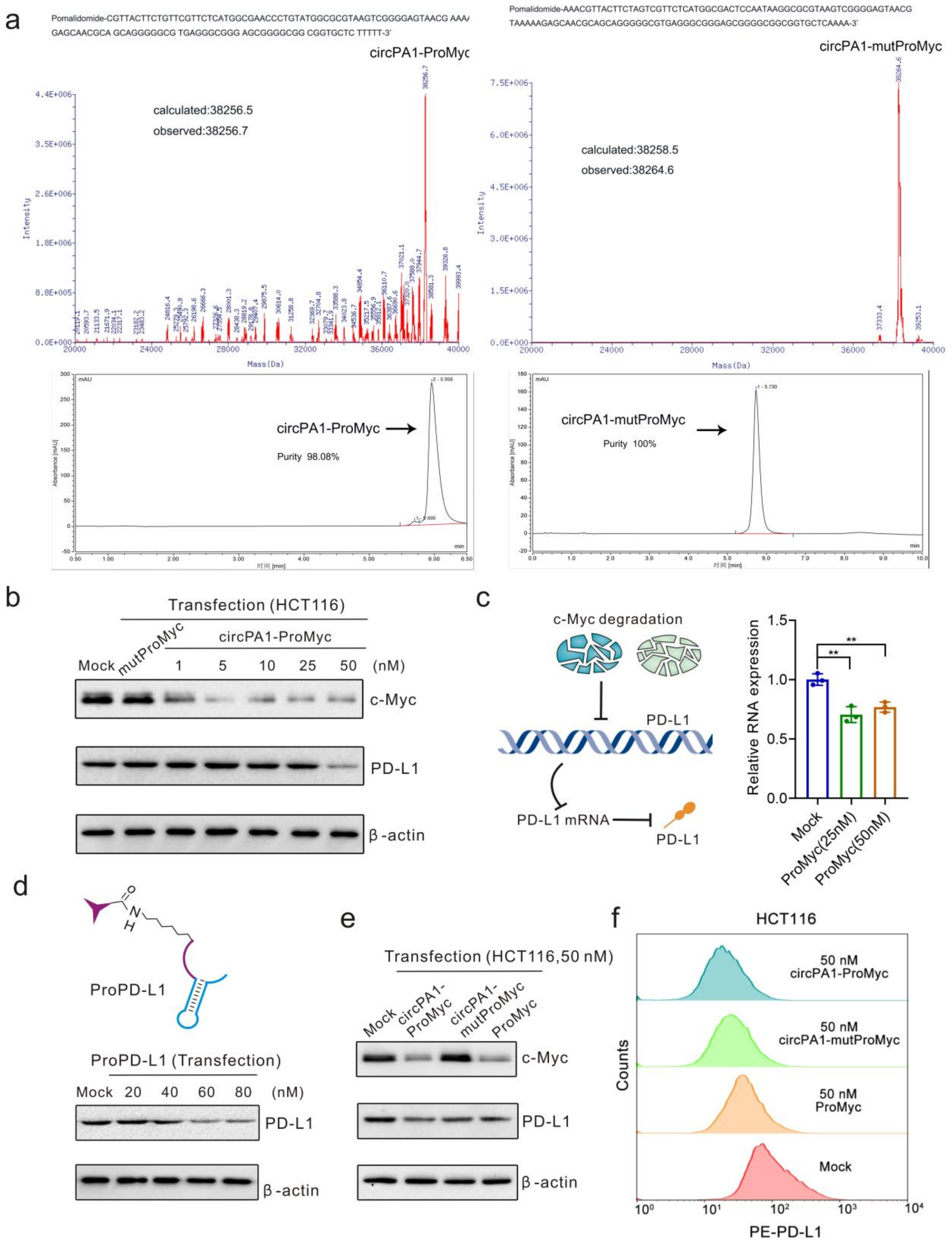

Supplementary Figure S9. The synthesis of circPA1-ProMyc and the antitumor potential of ProMyc.

a. Electrospray ionization mass spectrometry spectra of aminomodified circPA1-MA9C1 and circPA1-

MA9C1-pomalidomide conjugate (circPA1-ProMyc) and purification of circPA1-ProMyc by UPLC. Free DNA was eluted first and DNA-pomalidomide conjugate was eluted later. **b**. Representative western blot analysis of the c-Myc and PD-L1 in HCT116 cells transfected with different concentrations of circPA1-ProMyc for 24 h. **c**. The diagram of c-Myc degradation was beneficial to the down-regulation of PD-L1 and qRT-PCR analysis of the PD-L1 gene expression in HCT116 cells transfected with different concentrations of ProMyc for 48 h. **d**. The structure of ProPD-Ll and the western blot analysis of the PD-L1 in HCT116 cells treated with different concentrations of ProPD-Ll for 24 h. **e**. Representative western blot analysis of the c-Myc and PD-L1 in HCT116 cells with the indicated treatment for 48 h. The cells were transfected with DNA at 50 nM. **f**. Flow cytometry analysis of surface PD-L1 (sPD-L1) of HCT116 cells with the indicated treatment for 48 h. All the error bars indicate standard deviations (n= 3). All the *P* values were determined. $^{*}P < 0.05$; $^{**}P < 0.005$; $^{***}P < 0.001$.

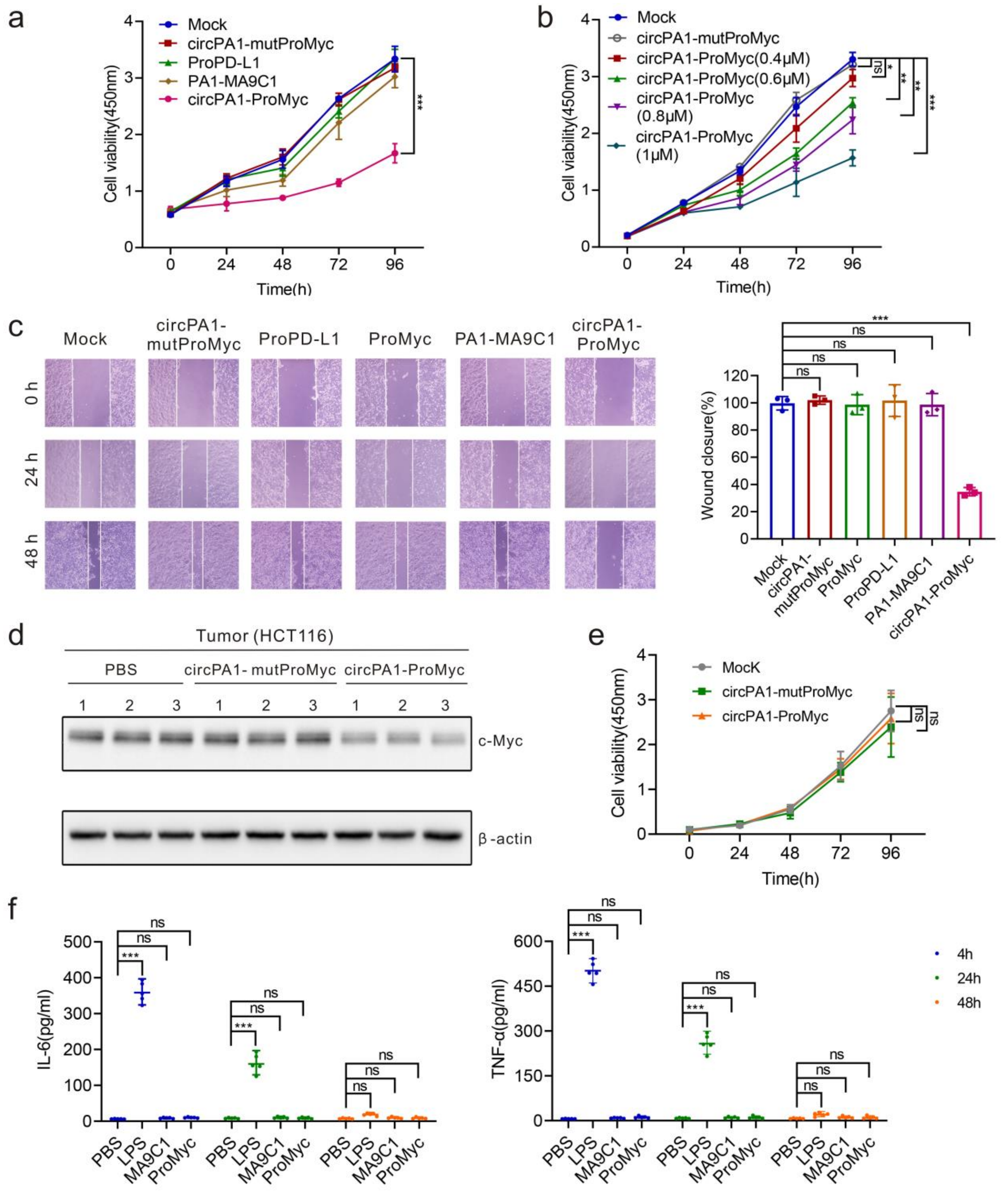

Supplementary Figure S10. The antitumor potential of circPA1-ProMyc. a. Identify the cell proliferation inhibition in HCT116 cells with the transfections of circPA1- ProMyc (50 nM) at the indicated times by CCK8 assay. **b.** The cell proliferation in HCT116 cells with the incubation of different concentration of circPA1- ProMyc at the indicated times by CCK8

assay. **c.** Wound healing images of HCT116 cells incubated with the indicated oligonucleotides (1 μM) and the wound closure was quantified. Mock: without treatment. **d**. Representative western blot analysis of c-Myc protein in tumor with the indicated treatment (n= 3 mice per group). **e**. Cell viability of HEK-293T cells after incubation with circPA1-ProMyc (1 μM). **f**. ELISA analysis of the immunogenicity of cicrPA1-ProMyc in mice. The mice were injected with 10 mg/kg oligonucleotides and 3 mg/kg LPS (n = 3 mice per group). Serum samples were collected at the indicated time after injection. All the error bars indicate standard deviations (n= 3). All the *P* values were determined. **P*＜0.05; ***P*＜0.005; ****P*＜0.001.

Supplementary Table S1 Mass spectroscopy analysis result of MA9-binding proteins. FC: the ratio of protein interacting with aptamer MA9 to those interacting with mutMA9C1.

Accession	Description	Score	Coverage (%)	Unique Peptides	PSMs	FC
P01106	Myc proto-oncogene protein	1641.69	30	15	890	288
P61626	Lysozyme C	1.95	14	2	3	198
Q03252	Lamin-B2	22.52	27	16	19	61
Q07065	Cytoskeleton-associated protein 4	36.18	13	4	11	59
P40227	T-complex protein 1 subunit zeta	10.28	17	6	8	59
P14866	Heterogeneous nuclear ribonucleoprotein L	173.71	31	12	131	58
O76021	Ribosomal L1 domain-containing protein 1	37.84	19	9	45	52
P50990	T-complex protein 1 subunit theta	4.22	6	3	3	49

Q13283	Ras GTPase-activating protein-binding protein 1	4.15	6	2	3	39
P52272	Heterogeneous nuclear ribonucleoprotein M	3.55	5	3	3	37
P10599	Thioredoxin	9.88	21	2	8	36
P31025	Lipocalin-1	3.51	13	2	4	29
Q9Y6M1	Insulin-like growth factor 2 mRNA-binding protein 2	1.82	8	3	4	27
Q9H223	EH domain-containing protein 4	7.69	8	4	4	25
P36578	60S ribosomal protein L4	92.96	26	9	74	23
Q16777	Histone H2A type 2-C	64.81	27	1	50	22
Q07666	KH domain-containing, RNA-binding, signal transduction-associated protein 1	4.99	10	3	5	22
P17844	Probable ATP-dependent RNA helicase DDX5	52.26	30	16	51	21
P02042	Hemoglobin subunit delta	2356.07	78	5	1120	21
Q9H6F5	Coiled-coil domain-containing protein 86	0	16	4	7	21
P31944	Caspase-14	2.11	10	2	4	21
P46087	Probable 28S rRNA (cytosine(4447)-C(5))-methyltransferase	13.38	12	9	20	20

Q562R1	Beta-actin-like protein 2	10.42	12	1	10	20
P31151	Protein S100-A7	2.17	11	1	2	20

Supplementary Table S2. The information of the DNA sequence used in the experiment. *Represents the phosphorothioate (PS) modification. Red T: Represents the NH2- modification.

Name	Sequences (5'-3')
MA9	CGTTACTTCTGTTCGTTCTCATGGCGAACCCTGTATGGCGCGTAAGTCGGGGAGTAACGTC TGCCTAGTTCCTTGTCTGATCTCCA
RS	TTCTGTTCGTTCTCATGCGCGTAAGTCGCGAACCCTGTATGGAGTAACGGGGGTCTGCCTA GTTCCTTGATCTCCAGTTAC
MA9C1	CGTTACTTCTGTTCGTTCTCATGGCGAACCCTGTATGGCGCGTAAGTCGGGGAGTAACG
MA9C1-1	CGTTACTTCTGTTCGTTCTCATGGTTTTTTTAGTAACG
MA9C1-2	GTTACTTCTGTTCGTTCTCATGGCGAACTTTTCGTAAGTCGGGGAGTAACG
MA9C1-3	GTTACTTCTGCCCTGTATGGCGCCGTAAGTCGGGGAGTAACG
MA9C1-L5	AAAAACGTTACTTCTGTTCGTTCTCATGGCGAACCCTGTATGGCGCGTAAGTCGGGGA GTAACG

MA9C1-L10	AAAAAAAAAACGTTACTTCTGTTCGTTCTCATGGCGAACCCTGTATGGCGCGTAAGTCGGGGAGTAACG
MA9C1-L15	AAAAAAAAAAAAAAACGTTACTTCTGTTCGTTCTCATGGCGAACCCTGTATGGCGCGTAAGTCGGGGAGTAACGAAAAA
mutMA9C1	GAAAGAGCTAGTCGAACTCATCACAACTCCAATAAGGCGCGTAAGTCGGGGACAATC
MA9C1-mut	CGTTAATTCTGTTCGTTCTCATGGCGAACCCTGTATGACACGTTTGACGGGGAGTAACG
stem-E box	GGGAGCACGTGGTTGCCACGTGGTTGGGTCAGTTCCCCAACCACGTGGCAACCACGTGCTCCCA
E box	TGGGAGCACGTGGTTGCCACGTGGTTGGG
	ACCCTCGTGCACCAACGGTGCACCAACCC
PA1	GAGCAACGCAGCAGGGGGCGTGAGGGCGGGAGCGGGGCGGCGGTGCTC
mutPA1	GAGCAACGCA GCAGGAAACGTGAGGGCGGGAGCGAAACGGCGGTGCTC
PA1-MA9C1	CGTTACTTCTGTTCGTTCTCATGGCGAACCCTGTATGGCGCGTAAGTCGGGGAGTAACGA*A*A*A*A*GAGCAACGCAGCAGGGGGCGTGAGGGCGGGAGCGGGGCGGCGGTGCTC
circPA1-MA9C1	AAACGTTACTTCTGTTCGTTCTCATGGCGAACCCTGTATGGCGCGTAAGTCGGGGAGTAACGTA*A*A*A*A*GAGCAACGCAGCAGGGGGCGTGAGGGCGGGAGCGGGGCGGCGGTGCTCAAAA
circPA1-mutMA9C1	AAACGTTACTTCTAGTCGTTCTCATGGCGACTCCAATAAGGCGCGTAAGTCGGGGAGTAACGTA*A*A*A*A*GAGCAACGCAGCAGGGGGCGTGAGGGCGGGAGCGGGGCGGCGGTGCTCAAAA
c-Myc ASO[6]	A*A*C*G*T*T*G*A*G*G*G*G*C*A*T*

Primer of c-Myc	CGGGTAGTGGAAAACCAGCCT
	TGCTGGTAGAAGTTCTCCTCCT
si-CRBN1	CUUAACGCGAUCUGCUCUGUUdTdT
	AACAGAGCAGAUCGCGUUAAGdTdT
si-CRBN2	CAGGAUAGUAAAGAAGCCAAAdTdT
	UUUGGCUUCUUUACUAUCCUGdTdT
Primer of CRBN	TGCTGTAAACAATGTCAAG
	AGGCATACCCAGGAAACC

References

[1] G. Yang, S. Zhang, Y. Wang, L. Li, Y. Li, D. Yuan, F. Luo, J. Zhao, X. Song, Y. Zhao, *Signal Transduct. Target. Ther.* **2022**, *7*.

[2] X. Wu, H. Liu, D. Han, B. Peng, H. Zhang, L. Zhang, J. Li, J. Liu, C. Cui, S. Fang, M. Li, M. Ye, W. Tan, *J. Am. Chem. Soc.* **2019**, *141*, 10760-10769.

[3] G. Yang, S. Zhang, W. Song, X. Bai, L. Li, F. Luo, Y. Cheng, D. Wang, Y. Wang, J. Chen, J. Zhao, Y. Zhao, *Small* **2023**.

[4] G. Y. Fatao Luo, Xia Bai, Deyu Yuan, Ling Li, Diyue Wang, Xiaoxiang Lu, Yiran Cheng, Yuchun Wang, Xu Song, Yongyun Zhao, *Cell Chem. Biol.* **2023**, *30*, 1-12.

[5] H. Han, A. D. Jain, M. I. Truica, J. Izquierdo-Ferrer, J. F. Anker, B. Lysy, V. Sagar, Y. Luan, Z. R. Chalmers, K. Unno, H. Mok, R. Vatapalli, Y. A. Yoo, Y. Rodriguez, I. Kandela, J. B. Parker, D. Chakravarti, R. K. Mishra, G. E. Schiltz, S. A. Abdulkadir, *Cancer Cell* **2019**, *36*, 483.

[6] R. Dhanasekaran, J. Park, A. Yevtodiyenko, D. I. Bellovin, S. J. Adam, A. Rajan, M. Gabay, H. Fernando, J. Arzeno, V. Arjunan, S. Gryanzov, D. W. Felsher, *Mol. Ther-Nucl. Acids* **2020**, *21*, 850-859.

Author Contributions

Conceptualization, Y.Z.; funding acquisition, Y.Z., X.S., andY.B.; investigation, Y.W. with the help of G.Y., X.Z., L.B., D.Y., D.G., Q.H., and L.Z.; data analysis, Y.Z., and Y.W.; technical guidance, X.S., Z.T., Y.B., Y.H., and Y.Y.; purification of DNA sequences, X.Z. and J.K.; writing original draft, Y.Z.; reviewing and editing, Y.Z.
